# *In vitro* assembly of the trehalose bi-enzyme complex with artificial scaffold protein

**DOI:** 10.3389/fbioe.2023.1251298

**Published:** 2023-08-29

**Authors:** Xiangyi Wang, Yi Jiang, Hongling Liu, Xinyi Zhang, Haibo Yuan, Di Huang, Tengfei Wang

**Affiliations:** ^1^ State Key Laboratory of Biobased Material and Green Papermaking (LBMP), Shandong Academy of Sciences, Qilu University of Technology, Jinan, Shandong, China; ^2^ Key Laboratory of Shandong Microbial Engineering, School of Bioengineering, Shandong Academy of Sciences, Qilu University of Technology, Jinan, Shandong, China

**Keywords:** trehalose, artificial scaffold protein, fusion enzyme, bi-enzyme complex, cascade enzyme reaction, substrate channel effect

## Abstract

**Introduction:** Trehalose is a significant rare sugar known for its stable properties and ability to protect biomolecules from environmental factors.

**Methods:** In this study, we present a novel approach utilizing a scaffold protein-mediated assembly method for the formation of a trehalose bi-enzyme complex. This complex consists of maltooligosyltrehalose synthase (MTSase) and maltooligosyltrehalose trehalohydrolase (MTHase), which work in tandem to catalyze the substrate and enhance the overall catalytic efficiency. Utilizing the specific interaction between cohesin and dockerin, this study presents the implementation of an assembly, an analysis of its efficiency, and an exploration of strategies to enhance enzyme utilization through the construction of a bi-enzyme complex under optimal conditions *in vitro*.

**Results and Discussion:** The bi-enzyme complex demonstrated a trehalose production level 1.5 times higher than that of the free enzyme mixture at 40 h, with a sustained upward trend. Compared to free enzyme mixtures, the adoption of a scaffold protein-mediated bi-enzyme complex may improve cascade reactions and catalytic effects, thus presenting promising prospects.

## Highlights

The specific interaction of cohesin–dockerin is applied to the other fields of non-cellulases.
Inspired by the configuration of cellulosomes, the assembled bi-enzyme complex can improve the cascade reaction and improve the catalytic effect compared with free enzyme mixtures.

## 1 Introduction

In nature, trehalose is a non-reducing disaccharide that is linked to the glucose residue by an α-1,1 glycoside bond (Trevelyan and Harrison, 1956; Mery et al., 2022). It is an excellent natural desiccant and preservative, as well as a new functional oligosaccharide (De Britto et al., 2021). Trehalose will form a unique protective membrane on the cell surface, effectively protecting the invariant inactivation of protein molecules, to maintain the normal life process and biological characteristics of living organisms in harsh environments characterized by high temperature, high cold, high osmotic pressure, and dry water loss (Camisasca et al., 2020; Chen et al., 2022). It has been known as the “sugar of life” in the scientific community. This unique functional feature enables trehalose to serve as an excellent active protective agent for protein drugs, enzymes, vaccines, and other biological products. The special biological characteristics of trehalose make it widely used in food, cosmetics, agriculture, and biological products (Eş et al., 2015; Taherimehr et al., 2022).

**FIGURE 1:**
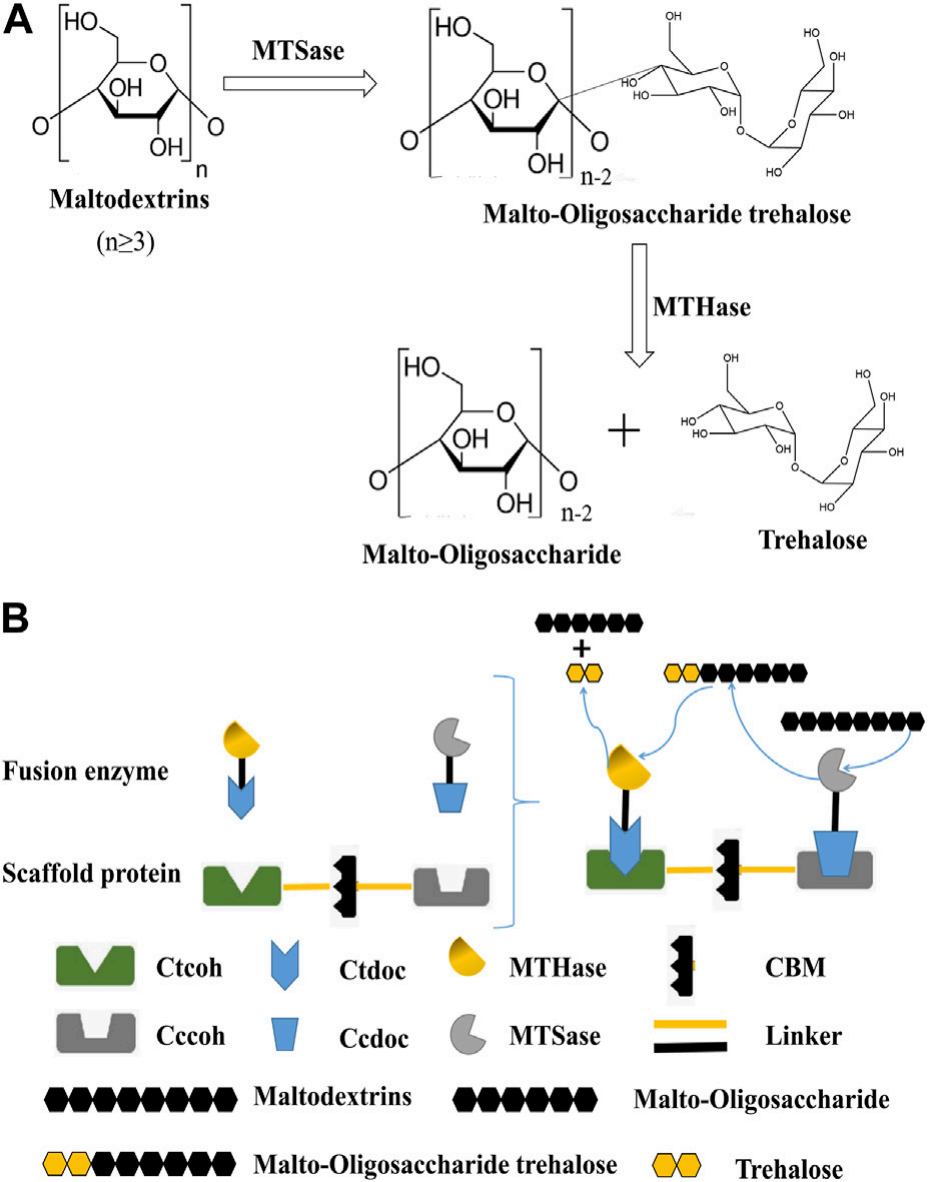
Preparation of trehalose by dual-enzyme catalysis. **(A)**: Reaction formula for the preparation of trehalose by MTSase and MTHase. N represents the degree of polymerization (DP) greater than or equal to 3. **(B)**: Construction of the trehalose bi-enzyme complex and schematic diagram of bi-enzyme catalysis. The scaffold protein and fusion enzymes were constructed according to the aforementioned methods, expressed in *E. coli*, mixed *in vitro*, and the recombinant fusion enzymes were assembled on the artificial scaffold protein by using the specific interaction between the cohesion protein and the dockerin protein to form a trehalose bi-enzyme complex. Cohesins are connected by a natural linker, and fusion enzymes are also connected by a linker.

At present, the production methods of trehalose mainly include microbial extraction, fermentation, and enzyme transformation (Cai et al., 2018). The technological route of microbial extraction has been mature, but it cannot be widely used in industrial production (Schiraldi et al., 2002). The production of trehalose by microbial fermentation has unique advantages but also has some disadvantages, such as the low conversion rate of trehalose and the complex composition of *Cupriavidus necator,* and engineering cyanobacteria were able to produce trehalose from CO_2_, but with time, the productivity of trehalose reduced, indicating that the cells were unable to fix CO_2_ as quickly as they formerly could due to light restrictions brought on by cell shading. Additionally, the genetic component of trehalose production and secretion needs to be better optimized (Ducat et al., 2012; Yue et al., 2020; Lowe et al., 2021). Enzyme conversion is presently the most potent method for the production of trehalose, which mainly uses maltose, glucose, starch, and dextrin as substrates and trehalose synthesis-related enzymes to produce trehalose. So far, there are five biosynthetic pathways of trehalose (De Britto et al., 2021). At present, the primary method of large-scale production of trehalose at home and abroad is to prepare trehalose by using the mixed catalytic starch liquefaction solution of maltooligosyltrehalose synthase (MTSase) and maltooligosyltrehalose trehalohydrolase (MTHase). It is completed in two steps under the joint action of MTSase and MTHase, as shown in Figure 1A. The MTSase is used to catalyze maltodextrin with a degree of polymerization (DP) greater than 3 to convert its reducing end α-1,4 connection key generation α-1,1 bond to obtain malto-oligosaccharide trehalose containing a trehalose group at the end. The MTHase-specific catalysis of α-1,1 bond trehalose releases a molecule of trehalose, and the reaction cycle continues until the DP is less than 3 to terminate the reaction (Flechard et al., 2010). When preparing trehalose with mixed catalysis of MTSase and MTHase, the enzymes in the catalytic system are in a free dispersion state, the proximity effect between enzymes is low, and the stoichiometric ratio of enzymes is difficult to be accurately controlled, resulting in an imbalance of proportion and waste of enzyme preparations, affecting the cascade catalytic efficiency of multiple enzymes (Zheng et al., 2023). Studies have shown that the substrate channel effect and synergistic mechanism are the main reasons for the significant improvement of the catalytic efficiency of multi-enzyme complexes after the self-assembly of multiple catalytic elements in some natural metabolic pathways (Iturrate et al., 2009). The substrate channel effect refers to the process in which the reaction intermediate is directly transferred from the first enzyme active site to the second enzyme active site during the cascade enzyme-catalyzed conversion process while ensuring the multi-enzyme catalytic system, the catalytic efficiency, and stability are improved (Shi et al., 2018; Jiang et al., 2021).

To improve the cascade catalytic efficiency of multiple enzymes, artificial scaffold protein-mediated enzyme complexes will be constructed. The scaffold proteins are defined as proteins that organize signal complexes by binding at least two signal enzymes together and facilitating their communication through proximity (Alexa et al., 2010; Srour et al., 2022). Currently, synthetic scaffolds are mainly used for soluble enzyme systems, and the most typical example is cellulosomes (Lamed et al., 1983; Bayer et al., 2008); a cellulose degradation-related protein complex was purified from *C. thermocellum* for the first time in 1983, which was proven to be a cellulosome. Notably, cellulosomes are divided into two parts: one part is a scaffold protein composed of multiple cohesins in different orders, quantities, and non-catalytic cellulose-binding modules (CBM), which can assemble functionally and bind multi-enzyme complexes to cellulose (Zverlov et al., 2008; Bule et al., 2016). The other part is the catalytic module composed of a range of cellulases connected with dockerins, which have a catalytic function. The two parts form a multi-enzyme complex through the interaction between dockerins and cohesins, which have specific interaction mechanisms between species and types (Gilmore et al., 2015; Ben Shabat et al., 2016). The cohesin–dockerin interaction is an effective way of assembling multi-enzyme complexes, which are constructed by fusing the required enzymes into the C-terminal of dockerins (Slutzki et al., 2015; Vera et al., 2021). Such nanomolar affinity between cohesin–dockerin modules makes it an ideal material for an *in vitro* skeleton system, which provides an important biological element for the orderly assembly of a multi-enzyme complex system. Based on the high-affinity and high specific interaction between cohesins and dockerins from natural cellulosomes, Meng et al. (2019) constructed four self-assembled synthetic enzyme complexes containing cellodextrin phosphorylase (CDP) and phosphoglucomutase (PGM) with different spatial organizations for generating bioelectricity from cellodextrin. The results showed that the *in vitro* biological system containing the optimal CDP–PGM enzyme complex exhibited significantly higher current density (3.35 times) and power density (2.14 times) than the corresponding biological system containing a mixture of free CDP and PGM (Meng et al., 2019). It was noted that the assembly with cellulosomes has a lot of strengths, such as small molecular weight, several binding sites, abundant types of dockerin, strong design ability, and so on (Gunnoo et al., 2018; Duarte et al., 2021). In the existing technology, the specific interaction of cohesin–dockerin is mostly applied for the degradation of cellulose, with relatively few applications for non-cellulase enzymes.

In this study, a bi-enzyme complex system was used to cascade functionally related enzymes to continuously catalyze the substrate and effectively improve the overall catalytic effect. Combined with the configuration of the cellulosome, the cohesin genes from different cellulosomes were chosen to obtain the artificial scaffold protein. Meanwhile, the dockerin genes derived from different cellulosomes would be fused with MTSase and MTHase genes, respectively, to obtain the fusion enzymes. The fusion enzymes were mixed with artificial scaffold protein based on the specific interspecific interaction between cohesins and dockerins, and the artificial scaffold protein-mediated trehalose bi-enzyme complex was self-assembled in the system (Figure 1B). In addition, the process of *in vitro* assembly was studied, and the conditions, efficiency, and structural morphology before and after assembly, as well as the enzymatic properties of the multi-enzyme complex after assembly, were analyzed, which laid a solid foundation for the research of scaffold protein-mediated multi-enzyme complexes in the future. The strategy of designing trehalose bi-enzyme complexes *in vitro* has a good utilization prospect so that the synergy between enzymes can be brought into full play through artificial design, and its enzyme activity can be doubled.

## 2 Materials and methods

### 2.1 Materials

All chemicals were purchased from Aobox Biotechnology (Beijing, China), Vazyme Biotech (Nanjing, China), and Sangon Biotech (Shanghai, China), unless stated otherwise. Restriction enzymes BamH I and Xho I were purchased from Thermo Fisher Scientific (United States). The plasmid purification kit was purchased from Sangon Biotech (Shanghai, China). The DNA sequences coding for the scaffold protein and fusion enzymes were synthesized by Sangon Biotech (Shanghai, China). *E. coli* strain BL21 (DE3) was used for the expression of recombinant plasmids and purchased from Vazyme Biotech (Nanjing, China). *Sulfolobus acidocaldarius* ATCC 33909 was used for the acquisition of MTSase and MTHase genes from MoBiTee. The culture medium mainly involved in this experiment includes Luria-Bertani (LB) medium and Terrific Broth (TB) medium. The LB medium was used as the culture medium for the activation culture of recombinant strains. The TB medium was used for fermentation and culture of scaffold proteins and fusion enzymes.

### 2.2 Construction of plasmids

The primers in Supplementary Table S1 were used to amplify DNA fragments on the corresponding template to prepare plasmids. All the plasmids used in this study are listed in Supplementary Table S2. Plasmid pUC57-ScafCCR, which has an expression cassette containing two cohesin modules from *Clostridium thermocellum* ATCC 27405 (*Ct*Coh), *Clostridium cellulolyticum* H10 (*Cc*Coh), and CBM module from *Clostridium thermocellum* ATCC 27405 (GenBank: OQ630934, OQ630935), respectively, were obtained from Sangon Biotech. The MTSase gene from *Sulfolobus acidocaldarius* ATCC 33909 (GenBank: OQ630936) and the dockerin gene *Cc*Doc from *C. cellulolyticum* H10 (GenBank: OQ630937) were fused to obtain plasmid pUC57-Sase-*Cc*Doc from Sangon Biotech. Meanwhile, the MTHase gene from *Sulfolobus acidocaldarius* ATCC 33909 (GenBank: OQ630939) and the dockerin gene *Ct*Doc from *C. thermocellum* ATCC 27405 (GenBank: OQ630938) were fused to obtain plasmid pUC57-Hase-*Ct*Doc from Sangon Biotech.

The scaffold gene ScafCCR was amplified by primers ScafCCR-F and ScafCCR-R using the synthesized pUC57-ScafCCR plasmid as a template. The catalytic module gene Sase-*Cc*Doc was amplified by primers Sase-*Cc*Doc-F and Sase-*Cc*Doc-R using the synthesized pUC57-Sase-*Cc*Doc plasmid as a template. The catalytic module gene Hase-*Ct*Doc was amplified by primers Hase-*Ct*Doc-F and Hase-*Ct*Doc-R using the synthesized pUC57-Hase-*Ct*Doc plasmid as a template. In addition, using the synthesized pUC57-Hase-*Ct*Doc plasmid as the template, the gene fragments of Hase and *Ct*Doc were amplified by primers Hase-F/Hase-R and *Ct*Doc-F/*Ct*Doc-R.

The target gene ScafCCR was connected with the linearized vector pET28a (+) digested by *BamH* Ⅰ and *Xho* Ⅰ by using a seamless cloning kit to obtain the product pET28a-ScafCCR, and then the product was transferred into the competent cell *E. coli* BL21 (DE3) after PCR verification was completed by universal primers pET28a-F and pET28a-R, and the recombinant strain *E. coli*/pET28a ScafCCR was constructed. The target gene Sase-*Cc*Doc was connected with a single fragment of the linearized vector pET28a digested by *BamH* Ⅰ and *Xho* Ⅰ according to the homology. The same target fragments *Ct*Doc and Hase completed the multi-fragment connection with the linearization vector pET28a according to the homologous sequence. After 30 min of exposure to constant temperature at 37°C, two connection products pET28a-Sase-*Cc*Doc and pET28a-*Ct*Doc-Hase were obtained, respectively. After being transformed into *E. coli* BL21 (DE3) competent cells, the recombinant strains *E. coli*/pET28a-Sase-*Cc*Doc and *E. coli/*pET28a-*Ct*Doc-Hase were constructed. The C-terminal of the scaffold gene and the fusion enzyme gene contains 6× His tag expression genes.

### 2.3 The expression and purification of scaffold protein and fusion enzymes

The recombinant strains *E. coli*/pET28a ScafCCR, *E. coli*/pET28a-Sase-*Cc*Doc, and *E. coli/*pET28a-*Ct*Doc-Hase were cultured in LB 1 day in advance, and single colonies were selected in LB culture medium 100 μg/mL kanamycin for activation culture at 37°C and 200 r/min. After shaking for 12 h, the seed solution was inoculated into a TB medium containing 100 μg/mL kanamycin at a ratio of 1% for fermentation culture, and when the OD_600_ reached approximately 1.5–2.0, 100 μM isopropyl-beta-D-thiogalactopyranoside (IPTG, final concentration) was added, and the culture was cultivated at 25 °C for another 12 h. The fermentation broth was centrifuged at 4°C, and the cell pellets were collected and suspended with 10 mM PBS (pH 7.4) containing 137 mM NaCl, 2.7 mM KCl, 1.4 mM KH_2_PO_4_, and 4.3 mM Na_2_HPO_4_ and broken by ultrasound on ice. The supernatant of a scaffold protein (ScafCCR) and fusion enzymes (Sase-*Cc*Doc, *Ct*Doc-Hase) were loaded into 12% SDS-PAGE to check the expression level of scaffold protein and fusion enzymes. In addition, the supernatant was separated and purified by ÄKTA™ start using the combination of His-tag and Ni^2+^ column, and the elution of labeled protein, miscellaneous protein, and other substances was realized according to different imidazole concentrations to obtain high-purity target protein. It was noted that high-purity target protein needs to be desalted and concentrated for not affecting the later assembly efficiency. Protein concentrations were determined using the Bradford method with bovine serum albumin as the standard.

### 2.4 Enzymatic activity assays

The supernatant enzyme activities of fusion enzymes Sase-*Cc*Doc and *Ct*Doc-Hase were determined according to the methods described elsewhere (Wang et al., 2001). The enzyme activity of the fusion enzyme Sase-*Cc*Doc was defined at 65°C and pH 5.5 as the amount of enzyme required to consume 1 μmol of maltose every 1 min. The enzyme activity of the fusion enzyme *Ct*Doc-Hase was defined as 75°C, pH 6.0, the amount of enzyme required to produce 1 μmol of trehalose every 1 min.

### 2.5 Optimization of conditions for Sase-*Cc*Doc and *Ct*Doc-Hase protein expression

To determine the optimal fermentation conditions for the fusion enzyme, we optimized the growth amount, IPTG addition, induction temperature, and induction time. When the OD_600_ of growth was 0.8–25, the enzyme activity of the crude enzyme solution of the fusion enzymes was measured to determine the ideal growth. The enzyme activity of the crude enzyme solution of the fusion enzyme was measured under the optimum growth conditions when IPTG (0.05–1 mM) was added to determine the optimal inducer supplementary level. Under the conditions of optimum growth and IPTG addition amount, the enzyme activities of the crude enzyme solution of the fusion enzymes were measured when the induction temperature was 20°C–37°C, respectively, to find the optimal induction temperature. The enzyme activity of the crude enzyme solution of the fusion enzymes was measured when the induction time was 7–48 h under the aforementioned ideal circumstances to establish the ideal induction time.

### 2.6 Bi-enzyme complex assembly onto scaffold protein

Under the optimal fermentation conditions, the purified, desalted, and concentrated fusion enzymes Sase-*Cc*Doc and *Ct*Doc-Hase were mixed and assembled with scaffold protein ScafCCR in equal molar concentrations, respectively, or at the same time. Native-PAGE (Darie et al., 2011) and SDS-PAGE were used to verify the interaction of cohesin–dockerin to further verify the effect of self-assembly. Six groups of samples (Sase-*Cc*Doc, *Ct*Doc-Hase, ScafCCR, *Ct*Doc-Hase + ScafCCR, Sase-*Cc*Doc + ScafCCR, and Sase-*Cc*Doc + *Ct*Doc-Hase + ScafCCR) were designed according to the fusion enzyme and artificial scaffold protein. An amount of 4–6 μg equal mole of each protein sample was fully mixed with sample buffer (10 mM PBS buffer, 10 mM CaCl_2_, pH = 7.4), and the protein was assembled by reacting in a thermostatic water bath at 37°C for 2 h. After the reaction is completed, Native-PAGE and SDS-PAGE are performed at low temperatures and pressure. Temperature and pH can affect the activity of fusion enzymes, and Ca^2+^ can promote the folding of docking proteins to form stable tertiary structures (Rincon et al., 2003). We investigated the effects of pH (2.5, 5.5, 7.4, and 9.0), the concentration of Ca^2+^ (1, 2, 5, 10, 20, and 25 mM), and temperature (25°C, 37°C, 55°C, and 70°C) on assembly efficiency.

The affinity pull-down experiment further verified the formation of the trehalose bi-enzyme complex, which was mainly based on the fact that the CBM domain contained in the designed artificial scaffold protein could adsorb microcrystalline cellulose. An amount of 4–6 μg of the four protein samples (Sase-*Cc*Doc, *Ct*Doc-Hase, ScafCCR, and Sase-*Cc*Doc + *Ct*Doc-Hase + ScafCCR) was thoroughly mixed with the reaction solution (10 mM CaCl_2_, 50 mM Acetate Buffer, and 2 mM EDTA), and then 10% of the cellobiose was added for reaction in a 37°C constant temperature water bath for 2 h. Then, the reaction samples were treated with microcrystalline cellulose for 1 h. The supernatant not combined with microcrystalline cellulose and the precipitated part combined with microcrystalline cellulose were obtained by centrifugation. To eliminate the influence of nonspecific binding, the precipitated part was resuspended twice with acetate buffer (added with 0.05% Tween). The unbound supernatant and the precipitated part combined after washing were mixed with protein loading buffer and boiled for SDS-PAGE verification. At the same time, the scaffold protein ScafCCR obtained by purified ultrafiltration was diluted by the concentration gradient. The obtained ScafCCR concentration gradient protein glue map was combined with ImageJ software to analyze the gray value, and the conversion from the gray value to an optical density (OD) was calculated through the formula to realize the quantification of the signal to draw the linear relationship between scaffold protein content and total OD. The assembly gray value of the fusion enzyme and scaffold protein was analyzed by ImageJ software. After signal quantification, the assembly efficiency of the fusion enzyme and scaffold protein was calculated by a linear relationship.

$$OD=\mathrm{lg}\left(\frac{255}{255-gray}\right).$$

Here, OD represents the optical density value, and gray represents the gray value.

### 2.7 Structural analysis, enzymatic properties, and detection of trehalose production of the trehalose bi-enzyme complex

After the assembly of the trehalose bi-enzyme complex, its structure, enzymatic properties, and ability to produce trehalose were explored. Molecular docking simulation and circular dichroism were used to analyze the structure of the successfully assembled trehalose bi-enzyme complex. In addition, the optimum temperature and pH of the trehalose bi-enzyme complex after assembly were studied. Meanwhile, HPLC was used to monitor the trehalose production capacity of the trehalose bi-enzyme complex.

The assembly of the trehalose bi-enzyme complex was simulated using AutoDockTools, and the docking calculation between proteins was realized by using the ZDOCK module (Pierce et al., 2011). ZDOCK is a rigid protein docking algorithm based on Fast Fourier Transform Correlation Technology, which can realize the translational and rotational space of the protein system, and the energy scoring function is used to score these docking configurations. Thus, the docking fraction between docking proteins *Cc*Doc, *Ct*Doc, and artificial scaffold protein ScafCCR was obtained. The structure of artificial scaffold protein and dockerin was simulated by a Swiss model and PyMOL (Supplementary Figure S1). The secondary structure of the bi-enzyme complex before and after assembly was analyzed by circular dichroism. Finally, APL data converter software is used to determine the percentage of the secondary structure of the sample.

In addition to analyzing the structure, the optimum temperature and pH of the assembled trehalose bi-enzyme complex were determined. The assembled trehalose bi-enzyme complex was measured at 40°C–90°C (interval of 5°C) to determine the enzyme activity, and it was defined that the enzyme activity of the bi-enzyme complex at the optimal temperature is 100%. The assembled trehalose bi-enzyme complex of PH was measured at 4.0–7.5 (interval of 0.5) to determine the enzyme activity, and it was defined that the enzyme activity of the bi-enzyme complex at the optimal PH is 100%. In addition, the stability of temperature and pH was explored. The enzyme activity of the trehalose bi-enzyme complex was defined as 100% at 0 h.

Purified fusion enzymes Sase-*Cc*Doc (95U/g) and *Ct*Doc-Hase (35U/g) were mixed and assembled with an appropriate amount of scaffold protein ScafCCR at the optimal pH and Ca^2+^ concentration. After the successful assembly of the trehalose bi-enzyme complex, its trehalose production effect needs to be tested. To ensure the full combination and assembly of fusion enzymes Sase-*Cc*Doc, *Ct*Doc-Hase, and scaffold protein ScafCCR, it is necessary to transfer the enzymes to a warm bath at 37°C for 1.5-2 h. Maltodextrin with a concentration of 200 mg/mL was used as the substrate and transformed at 60°C for 60 h. The production of trehalose in the mixture of free enzyme and bi-enzyme complex was monitored by HPLC, and the data were statistically analyzed by GraphPad Prism software (SPSS). A mixture of free enzyme MTSase and MTHase was used as the control group to compare the trehalose production efficiency of free enzyme mixture and bi-enzyme complex in a certain period.

## 3 Results

### 3.1 Expression of the artificial scaffold protein and fusion enzymes

**FIGURE 2:**
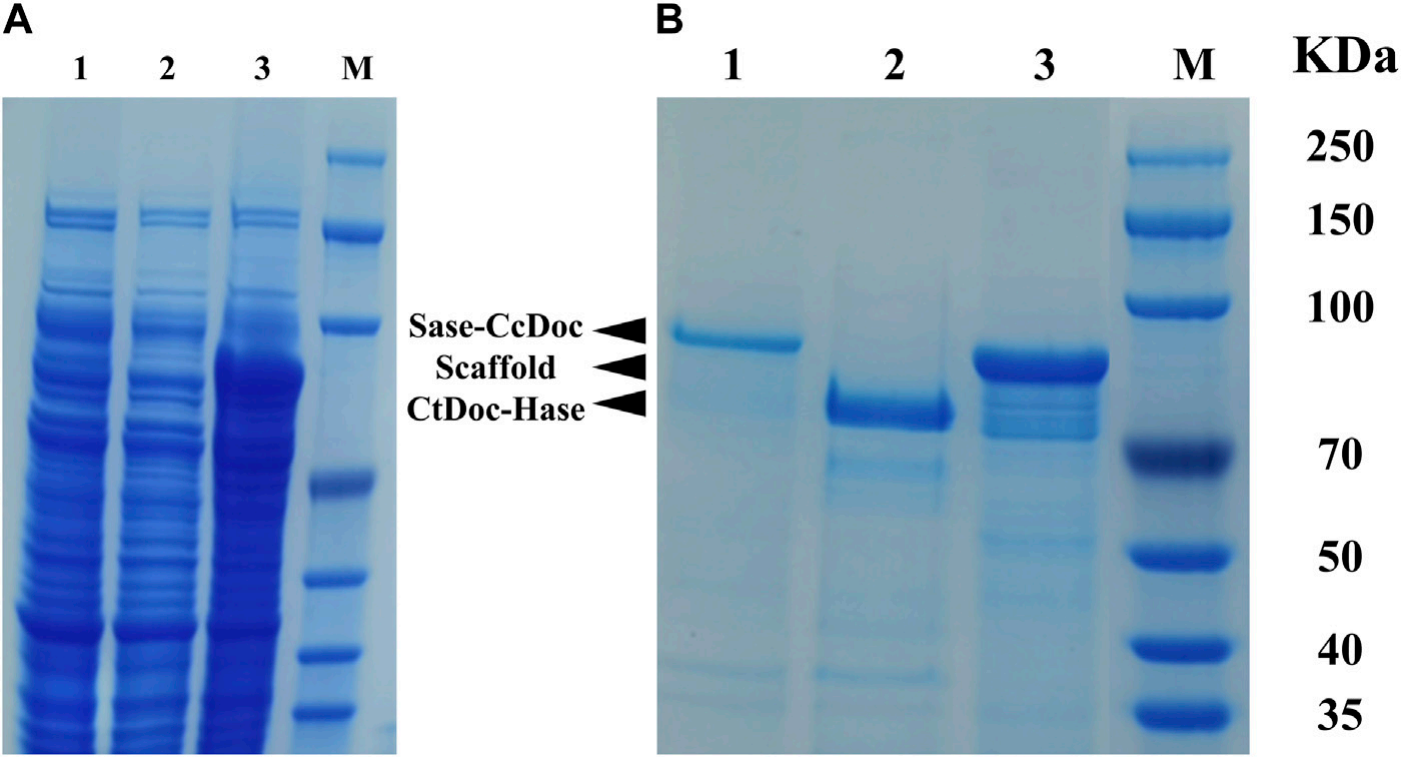
SDS-PAGE analysis of the *E. coli* cell extracts containing the supernatant of ScafCCR, Sase*-Cc*Doc, and *Ct*Doc*-*Hase before and after purification. **(A)**: Electrophoresis of the crude enzyme solution before purification, lane 1: Sase-*Cc*Doc; lane 2: ScafCCR; lane 3: *Ct*Doc*-*Hase. **(B)**: Electrophoresis of purified target protein, lane 1: Sase-*Cc*Doc; lane 2: ScafCCR; lane 3: *Ct*Doc*-*Hase. M represents the protein marker.

The supernatants of ScafCCR, Sase*-Cc*Doc, and *Ct*Doc*-*Hase before and after purification were loaded into 12% SDS-PAGE to check the expression level of scaffold protein and fusion enzymes, as shown in Figure 2. Before protein purification, the target protein cannot be seen from lanes 1–3 in Figure 2A, which represent the crude enzyme solutions Sase-*Cc*doc, *Ct*doc-Hase, and ScafCCR, respectively. However, after protein purification, the target protein can be seen from lanes 1–3 in Figure 2B. The size of the single band observed in lane 1 is consistent with the theoretical value of 93 kDa for the fusion enzyme Sase*-Cc*Doc. Lane 2 is the band of the fusion enzyme *Ct*Doc*-*Hase, and the theoretical value is consistent with the actual band, with a size of 74 kDa. The target band with a size of approximately 77 kDa can be observed from lane 3, which is consistent with the theoretical value of ScafCCR. The bands of SDS-PAGE indicated that the scaffold protein and fusion enzymes were successfully constructed and expressed, as expected. For subsequent assembly experiments, each protein was measured at a protein concentration and stored. The protein concentrations of the scaffold protein and fusion enzymes were 1,150.4 μg/mL, 342.84 μg/mL, and 283.93 μg/mL, respectively.

### 3.2 Optimization of conditions for fusion enzyme protein expression in *E. coli*

After the recombinant Sase*-Cc*Doc and *Ct*Doc*-*Hase were successfully expressed, the optimal fermentation conditions were selected by optimizing the growth amount, IPTG addition amount, induction temperature, and induction time in Supplementary Figure S2. When the growth OD_600_ value of *E. coli*/pET28a Sase*-Cc*Doc strain reached 3.5 (the biomass concentrations was approximately 2.8 × 10^9^/mL), the crude enzyme activity of the broken fermentation broth was approximately 5.5 U/mL (Supplementary Figure S2A). The OD value of *E. coli*/pET28a *Ct*Doc*-*Hase strain reached 3.0 (the biomass concentration was approximately 2.4 × 10^9^/mL), and the crude enzyme activity was approximately 7.6 U/mL (Supplementary Figure S2B). On the premise of determining the optimal growth amount, the additional amount of the IPTG inducer of *E. coli*/pET28a Sase-*Cc*Doc and *E. coli*/pET28a *Ct*Doc*-*Hase had the best effect at 0.1–0.2 mM; especially at 0.1 mM, the crude enzyme activities were 7.5 U/mL and 9.1 U/mL, respectively, as shown in Supplementary Figures S2C, D. Under the conditions of the best growth amount and the best addition of IPTG inducer, the two recombinant strains *E. coli*/pET28a Sase*-Cc*Doc and *E. coli*/pET28a *Ct*Doc*-*Hase were fermented at 20°C–37°C. It can be seen from Supplementary Figure S2E that the strain grows well within 25°C–30°C and is easier to grow at a lower temperature, while the strain can easily produce enzymes, and the enzyme activity of the fermentation broth is the highest at 27°C, and the crude enzyme activity can reach approximately 10.7 U/mL and 12.3 U/mL, respectively (Supplementary Figure S2F). Under the aforementioned optimization conditions of the growth amount, IPTG addition, and induction temperature, the optimal induction time of recombinant strains *E. coli*/pET28a Sase-*Cc*Doc and *E. coli*/pET28a *Ct*Doc-Hase is explored in Supplementary Figures S2G, H. Induction time can affect protein expression, and it can be seen that when the temperature is changed to 27°C and the induction time is controlled to 16 h, the crude enzyme activities of the two recombinant strains are approximately 12.9 U/mL and 15.8 U/mL, respectively. Through the optimization of the growth amount, IPTG addition amount, induction temperature, induction time, and other factors, the optimal fermentation conditions were selected, which laid a foundation for the purification and assembly of the fusion enzymes Sase-*Cc*Doc and *Ct*Doc*-*Hase. The MTSase and MTHase were cascade enzymes, and to accurately determine the amount of MTSase and MTHase in the fusion enzyme and ensure the correct use of enzyme units, the specific enzyme activities of the fusion enzymes Sase*-Cc*Doc and *Ct*Doc*-*Hase were measured with maltose hexasaccharide and tetrasaccharide as substrates, respectively. As shown in Supplementary Table S3, the specific enzyme activities of Sase*-Cc*Doc and *Ct*Doc*-*Hase were 178.64 U/mg and 265.27 U/mg, respectively. Compared with the previously constructed recombinant strain *E. coli*/pET28a Hase*-Ct*Doc, no obvious enzyme activity was determined.

### 3.3 Assembly of a bi-enzyme complex on scaffold protein

**FIGURE 3:**
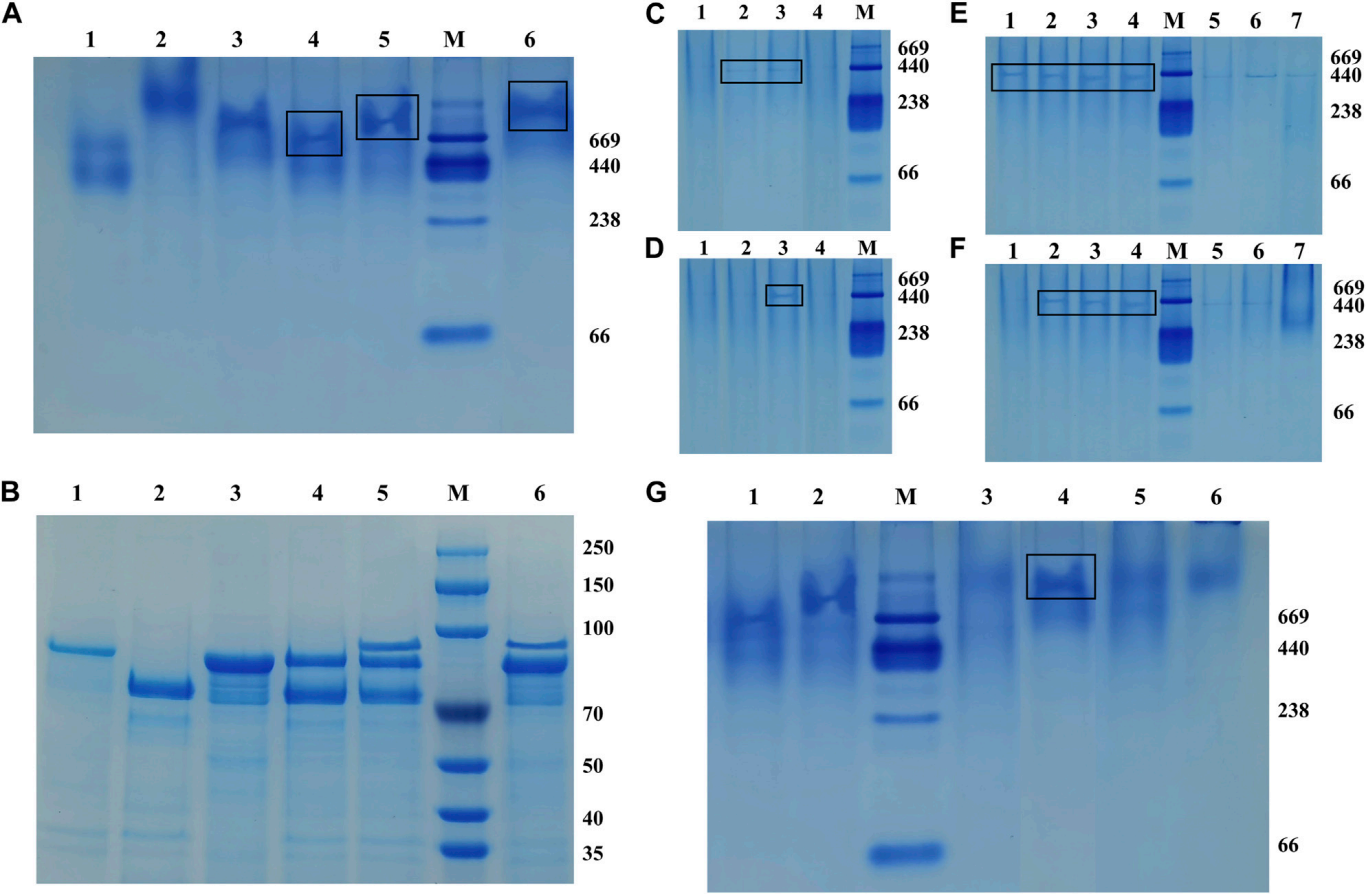
Native-PAGE and SDS-PAGE were used to compare the interaction between cohesins and dockerins, and different assembly conditions of the optimal pH, Ca^2+^ concentration, and temperature were studied. **(A)**: Native-PAGE electrophoresis was composed of Sase*-Cc*Doc (lane 1), *Ct*Doc*-*Hase (lane 2), ScafCCR (lane 3), ScafCCR + *Ct*Doc*-*Hase (lane 4), ScafCCR + Sase*-Cc*Doc + *Ct*Doc*-*Hase (lane 5), and ScafCCR + Sase*-Cc*Doc (lane 6). **(B)**: SDS-PAGE electrophoresis compared with Native-PAGE. **(C)**: Native-PAGE electropherograms of different PH values of ScafCCR + Sase*-Cc*Doc. **(D)**: Native-PAGE electropherograms of different PH values of ScafCCR + *Ct*Doc*-*Hase (lanes 1–4: PH = 2.5, 5.5, 7.4, and 9.0). **(E)**: Native-PAGE electropherograms of different Ca^2+^ concentrations of Sase*-Cc*Doc. **(F)**: Native-PAGE electropherograms of different Ca^2+^ concentrations of ScafCCR + *Ct*Doc*-*Hase. Ca^2+^ concentration: 1 mM, 2 mM, 5 mM, 10 mM, 15 mM, 20 mM, and 25 mM, successively (lanes 1–7). **(G)** Native-PAGE electropherograms of different temperatures of ScafCCR + Sase*-Cc*Doc + *Ct*Doc*-*Hase (lane 1: Sase*-Cc*Doc; lane 2: *Ct*Doc*-*Hase; lanes 3–6: T = 25°C, 37°C, 55°C, and 70°C).

The recombinant fusion enzymes were assembled on the artificial scaffold protein by using the specific interaction between the dockerins and cohesins in the scaffold protein to form a trehalose bi-enzyme complex. As shown in Figure 3, Native PAGE and SDS-PAGE electrophoresis results of the self-assembled trehalose bi-enzyme complex were compared, and the ScafCCR of lane 3 in Figure 3A was used as a reference for assembly analysis. It was observed that the assembly protein band of ScafCCR and *Ct*Doc*-*Hase was single and decreased in lane 4. In lane 6, the assembly band of ScafCCR and Sase*-Cc*Doc was single and increased. Observing lane 5, it was found that the trehalose bi-enzyme complex protein band formed by the assembly of Sase-*Cc*Doc, *Ct*Doc*-*Hase, and ScafCCR was single and between the protein bands of the scaffold assembled by the two fusion enzymes. Meanwhile, comparing lanes 4–6 in Figures 3A, B showed that the single band had completed the docking of dockerins and cohesins, forming a new whole. Therefore, it suggested that the designed and constructed fusion enzymes Sase-*Cc*Doc and *Ct*Doc*-*Hase can be completely assembled with the artificial scaffold protein ScafCCR and finally form a trehalose bi-enzyme complex.

To further study the optimum conditions of assembly, the pH, Ca^2+^ concentration, and temperature of the assembly between dockerin and cohesin were explored. Temperature and pH can affect the activity of fusion enzymes, and Ca^2+^ can promote the folding of docking proteins to form stable tertiary structures. The results showed that there were obvious bands when the pH of lanes 2–3 in Figure 3C was 5.0 and 7.4, while there were obvious bands when the pH of lane 3 in Figure 3D was 7.4. In other cases, there were no obvious bands, indicating that the optimal pH for *Cc*Coh-CcDoc was between 5.0 and 7.4, while the optimal pH for *Ct*Coh-*Ct*Doc was 7.4. In the neutral environment, it can be seen that the protein bands are obvious when the Ca^2+^ concentration is 1–10 mM from lanes 1–4 as in Figure 3E, and when the Ca^2+^ concentration is 2–10 mM from lanes 2–4 as in Figure 3F, the protein bands are easily observed in the neutral environment. In addition, the bands in this interval have little change, indicating that the self-assembly effect of the trehalose bi-enzyme complex is better at the concentration of 2–10 mM Ca^2+^. For assembly at the concentration of 15–25 mM Ca^2+^ of lanes 5–7 in Figures 3E, F, it is obvious from the degree of protein band change that the docking effect between dockerins and cohesins gradually gets worse, resulting in incomplete self-assembly. Based on the aforementioned conditions, taking the assembly of the fusion enzyme Sase-*Cc*Doc and the scaffold protein scafCCR as an example, the optimal temperature of docking between dockerins and cohesins was explored. It can be observed that the protein bands of the complex formed by the assembly at 37°C are clearer and undivided than those formed at other temperatures in Figure 3G, indicating that the assembly effect of the bi-enzyme complex is better at 37°C, and the docking between dockerin and cohesin is more complete. Through the study of module assembly under pH, temperature, and Ca^2+^ concentration, it was found that the trehalose bi-enzyme complex is controlled in a neutral environment, Ca^2+^ concentration is 2–10 mM, and the effect of assembly *in vitro* at 37°C is the best.

**FIGURE 4:**
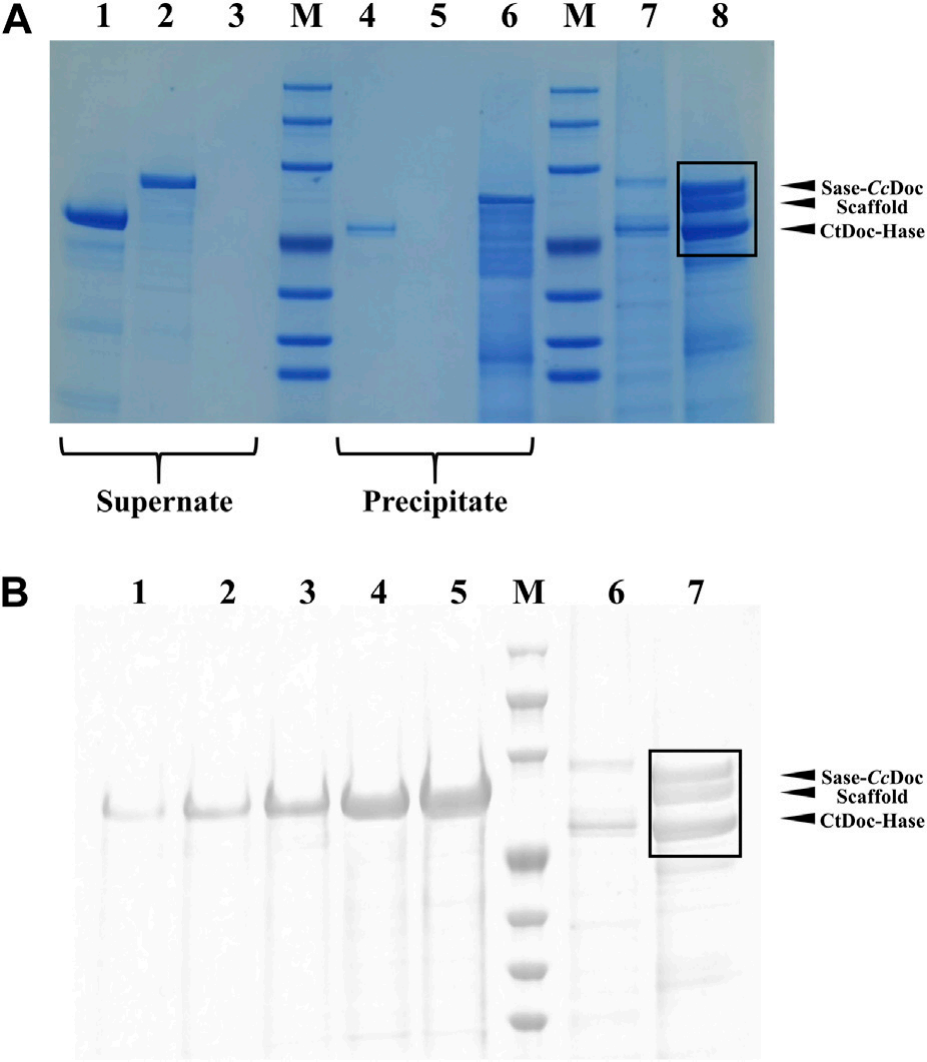
SDS-PAGE verification diagram of affinity pull-down experiment and the assembly efficiency of the trehalose bi-enzyme complex. **(A)**: Verification of the formation of the trehalose bi-enzyme complex by the affinity pull-down test (Lane 1–3: the supernatant of *Ct*Doc-Hase, Sase*-Cc*Doc, and ScafCCR; Lane 4–6: precipitation part of *Ct*Doc-Hase, Sase*-Cc*Doc, and ScafCCR; Lane 7–8: the supernatant and precipitation of ScafCCR + Sase*-Cc*Doc + *Ct*Doc-Hase). **(B)**: ImageJ software processing diagram for assembly efficiency of the trehalose bi-enzyme complex. (Lane 1–5: represent the concentration of scaffold protein, 0.02519, 0.05038, 0.10077, 0.21533, and 0.40306 μg/μL, successively, Lane 6: represents the supernatant and precipitation of ScafCCR + Sase*-Cc*Doc + *Ct*Doc-Hase).

In addition, we further verified the formation of the trehalose bi-enzyme complex by the affinity pull-down experiment. As shown in Figure 4A, comparing the supernatant part with the precipitation part of lanes 1–6, it can be observed that the protein bands of fusion enzymes *Ct*Doc-Hase and Sase-*Cc*Doc are present in the supernatant, indicating that they are not combined with microcrystalline cellulose. There was a band of lane 4 in the precipitation part that may be due to the incomplete centrifugation, resulting in a small amount of protein remaining in the precipitation. The remaining two unassembled fusion enzymes can be observed in the supernatant of the trehalose bi-enzyme complex from lane 7, and three protein bands can be observed in the precipitation part of the trehalose bi-enzyme complex from lane 8, indicating that the scaffold protein ScafCCR containing CBM assembled with the fusion enzymes *Ct*Doc-Hase and Sase-*Cc*Doc to form a bi-enzyme complex and closely adsorbed with microcrystalline cellulose, which verified the formation of the trehalose bi-enzyme complex.

Meanwhile, the assembly efficiency of the trehalose bi-enzyme complex was studied. According to the concentration gradient map of ScafCCR, the corresponding gray value was obtained through image analysis by ImageJ software of lanes 1–5 in Figure 4B. Lanes 6–7 in Figure 4B were the protein bands separated from the samples of the supernatant part and the precipitation part of the trehalose bi-enzyme complex in the affinity pull-down experiment. Due to the residual unassembled fusion enzyme in the supernatant during the assembly process, the assembly part of lane 7 is analyzed by ImageJ software. Supplementary Table S4 shows the relevant gray values and total OD values obtained; according to the linear relationship equation, the protein contents of the fusion enzymes Sase-*Cc*Doc and *Ct*Doc-Hase assembled on the scaffold protein in lane 7 were approximately 3.51 μg and 3.29 μg, respectively. Because the protein addition of Sase-*Cc*Doc and *Ct*Doc-Hase was 5 μg, the assembly and docking efficiency of scaffold protein ScafCCR and fusion enzymes Sase-*Cc*Doc and *Ct*Doc-Hase were approximately 70.16% and 65.94%, respectively.

### 3.4 Structural analysis, enzymatic properties, and detection of trehalose production of the trehalose bi-enzyme complex

**FIGURE 5:**
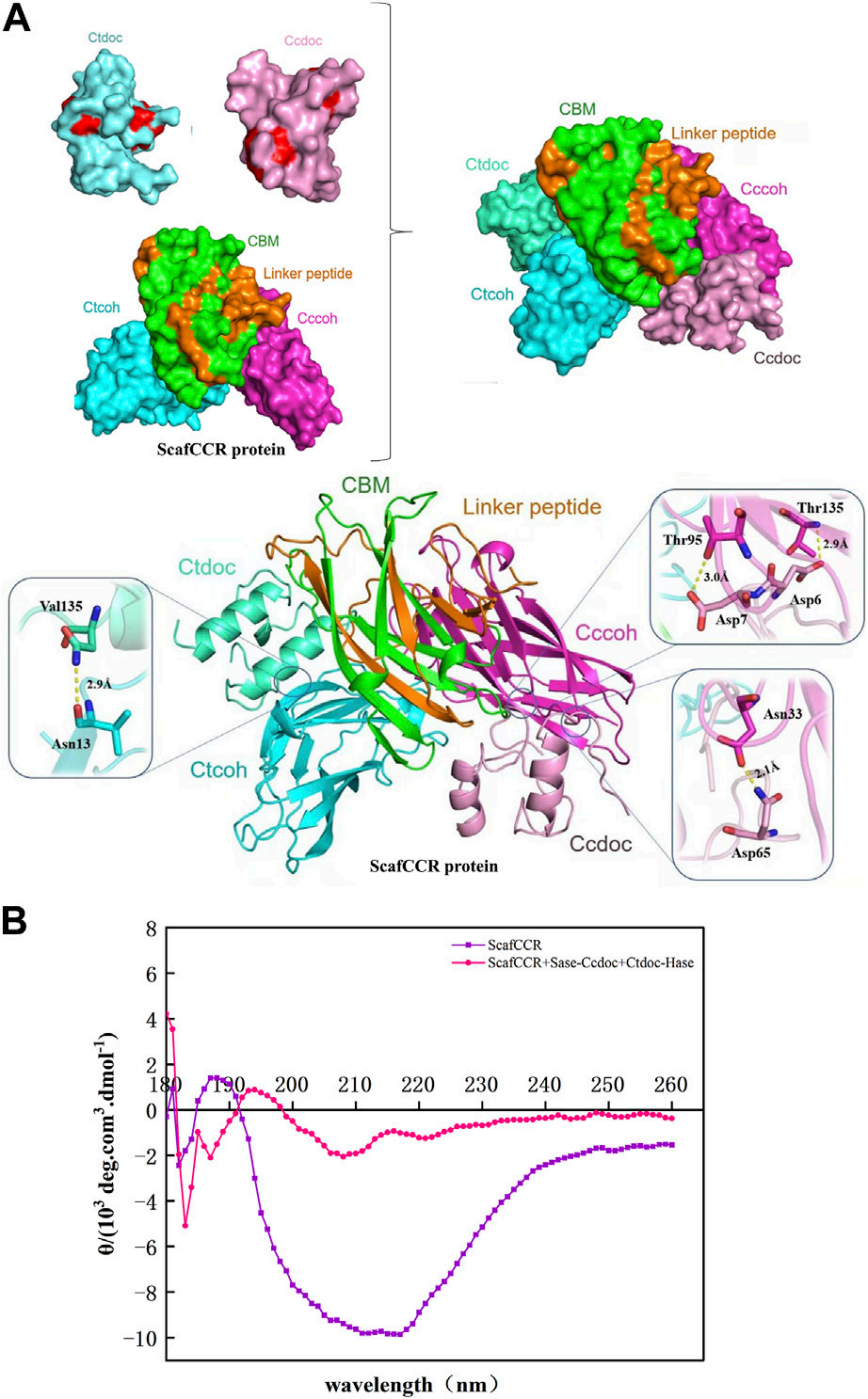
Structural analysis of the trehalose bi-enzyme complex. **(A)**: Docking simulation structure diagram of dockerin–cohesin. The amino acids Thr 95 and Thr 135 on *Cc*Coh in ScafCCR and Asp 7 and Asp 6 on *Cc*Doc form two hydrogen bonds, respectively, and the action distances between them are 3.0 Å and 2.9 Å, respectively; the amino acid Asn 33 on *Cc*Coh forms a hydrogen bond with the amino acid Asp 65 of *Cc*Doc, and the action distance is 2.1 Å. Amino acid Asn 13 on *Ct*Coh forms a hydrogen bond with amino acid Val 135 on *Ct*Doc, and the action distance is 2.9 Å. **(B)**: Docking simulation structure diagram of dockerin–cohesin by circular dichroism. The unassembled ScafCCR has a positive peak at 188 nm and a negative peak at 216 nm. After assembly, the scaffold protein showed a negative peak at 221 nm and 208 nm and a positive peak at 193 nm.

We further analyzed the structure, enzymatic properties, and trehalose production capacity of the assembled trehalose bi-enzyme complex. To fully understand the structure of the bi-enzyme complex, the ZDOCK module was used to calculate the docking fraction of ScafCCR with fusion enzymes Sase-*Cc*Doc and *Ct*Doc-Hase. The structure of artificial scaffold protein and dockerin was simulated by a Swiss model and PyMOL in Supplementary Figure S1. Figure 5 shows the docking simulation diagram between dockerins and cohesins. The dockerins *Cc*Doc and *Ct*Doc in the fusion enzyme interact with the cohesins *Cc*Coh and *Ct*Coh in ScafCCR, respectively, to form a new structure of a bi-enzyme complex. It can be observed from Figure 5A that the amino acids Thr 95 and Thr 135 on *Cc*Coh in ScafCCR and Asp 7 and Asp 6 on *Cc*Doc form two hydrogen bonds, respectively, and the action distances between them are 3.0 Å and 2.9 Å, respectively. At the same time, another action site of *Cc*Doc*-Cc*Coh is shown in which the amino acid Asn 33 on *Cc*Coh forms a hydrogen bond with the amino acid Asp 65 of *Cc*Doc, and the action distance is 2.1 Å. The details on the left of Figure 5A show the interaction between *Ct*Coh and *Ct*Doc in ScafCCR. Amino acid Asn 13 on *Ct*Coh formed a hydrogen bond with amino acid Val 135 on *Ct*Doc, and the action distance is 2.9 Å. The two groups were scored by the ZDOCK docking algorithm. The docking score of *Cc*Doc in the fusion enzyme and *Cc*Coh in the scaffold protein was 93.46%, and the docking score of *Ct*Doc in the fusion enzyme and *Ct*Coh in the scaffold protein was 91.34%. Meanwhile, the secondary structure of the trehalose bi-enzyme complex was determined by circular dichroism (CD). As shown in Figure 5B, the CD spectrum of the scaffold protein before and after assembly was drawn. Through analysis, it was found that the scaffold protein ScafCCR before and after the assembly had significant structural differences, and the unassembled ScafCCR had a positive peak at 188 nm and a negative peak at 216 nm. Calculated with APL data converter software, we show the percentage of the secondary structure in Table 1, from which we can know that the unassembled scaffold protein ScafCCR main chain is based on β-sheet dominated secondary structure, which is also consistent with the secondary structure observed in the aforementioned molecular simulation. The CD spectrum in Figure 5B showed negative peaks at 221 nm and 208 nm and a positive peak at 193 nm, which was after assembly. This demonstrated the presence of the scaffold protein in α-helical conformation after assembly. The interaction between dockerin and cohesin may have caused the peak value of the scaffold protein to decrease after assembly. The two repeated segments of dockerins recognized the polar amino acid sites outside the corresponding cohesins’ double lamellar structure and connected with it to enhance the order of the protein, resulting in the existence of the α-helix conformation in the assembled trehalose bi-enzyme complex. From the aforementioned simulated molecular docking, the effect of dockerins can be observed in the α-helical secondary structure, when dockerins and cohesins were butted to form a strong hydrogen bond, changing the secondary structure of the scaffold protein, which is why there were two configurations after assembly.

**TABLE 1:** Percentage of the secondary structure before and after scaffold protein assembly.

Protein	α-helix (%)	β-strand (%)	β-turn (%)	Random coil (%)
ScafCCR	13.6	38.7	20.8	29.8
ScafCCR + Sase-*Cc*Doc + *Ct*Doc-Hase	66	18.6	25	29.7

**FIGURE 6:**
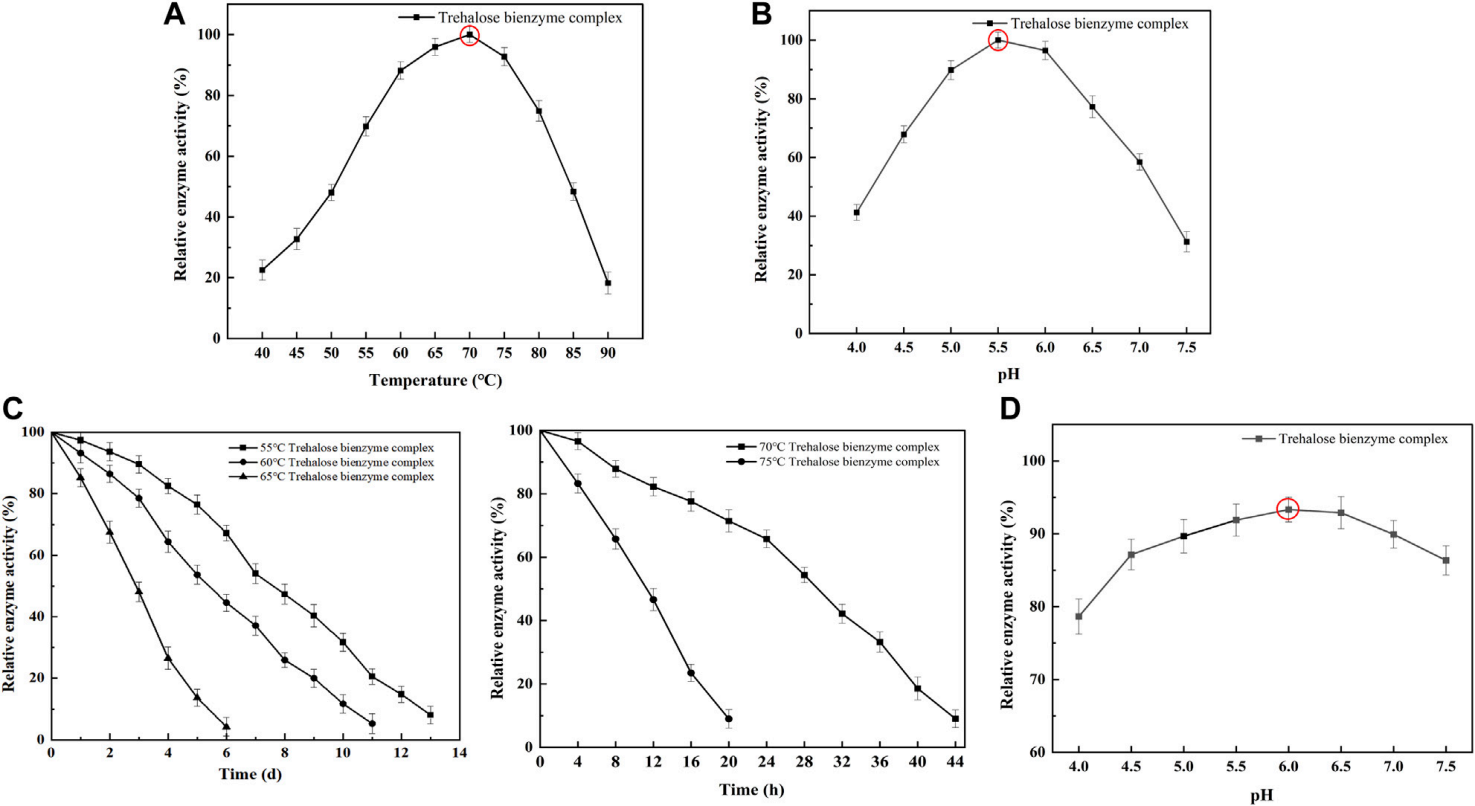
Enzymatic properties of the trehalose bi-enzyme complex. **(A)**: Optimum temperature of the trehalose bi-enzyme complex after assembly; the assembled trehalose bi-enzyme complex was placed at 40°C–90 C with an interval of 5°C to determine the enzyme activity. **(B)**: Optimum PH of the trehalose bi-enzyme complex after assembly; the enzyme activity of the assembled trehalose bi-enzyme complex was measured at pH 4.0–7.5 with an interval of 0.5. **(C)**: Temperature stability of the trehalose bi-enzyme complex; the assembled trehalose bi-enzyme complex was treated in a warm bath at 55, 60, 65, 70, and 75 °C. **(D)**: pH stability of the trehalose bi-enzyme complex; the enzyme activity was measured after the complex was treated at pH 4.0–7.5 (interval 0.5) for 24 h.

To understand the enzymatic properties of the assembled trehalose bi-enzyme complex, the optimum temperature, the optimum pH, and the stability of temperature and pH were investigated. As shown in Figure 6A, the optimum temperature of the assembled trehalose bi-enzyme complex is 70°C. When the temperature was lower than 70°C, the relative enzyme activity showed an increasing trend. Once the temperature is exceeded, the relative enzyme activity will decrease significantly. For the assembled trehalose bi-enzyme complex, the temperature will not only affect its enzyme activity but also affect its efficiency. When the optimum temperature is less than 70°C, the enzyme efficiency will be lower than that at the maximum efficiency with the decrease in activity. Similarly, when the temperature exceeds the optimum, the enzyme activity will decrease or even inactivate. Figure 6B showed that the optimum pH of the trehalose bi-enzyme complex was 5.5. When the pH was in the range of 4.0–5.5, the relative enzyme activity increased, and when the pH was 6.0, the relative enzyme activity can be maintained at more than 90%, but when the pH exceeded 6.0, the enzyme activity decreased rapidly. The temperature and pH stability of the trehalose bi-enzyme complex were analyzed, and the initial enzyme activity was 100%. The temperature stability of the trehalose bi-enzyme complex is shown in Figure 6C. With the continuous increase in temperature, the stability of the bi-enzyme complex was also relatively poor. When the temperature was 55°C, the half-life of the bi-enzyme complex was 7.5 d. When the temperature was maintained at 60°C, the half-life was shortened to 5 d. When the temperature was 65°C, the half-life changed to 2.5 d. When the temperature continued to increase to 70°C and 75°C, the half-life of the bi-enzyme complex decreased to 28 h and 9 h, respectively. Figure 6D shows the pH stability of the trehalose bi-enzyme complex. When the enzyme activity was measured after 24 h under different pH values, it was found that when the pH was 4.0–6.0, the relative enzyme activity of the bi-enzyme complex gradually increased with the increase of pH, and the stability was the worst when the pH was 4.0. When the pH was higher than 6.0, the relative enzyme activity of the bi-enzyme complex gradually decreased. Only when the pH is maintained between 5.5 and 6.5, the relative enzyme activity of the trehalose bi-enzyme complex can be maintained at approximately 90%.

**FIGURE 7:**
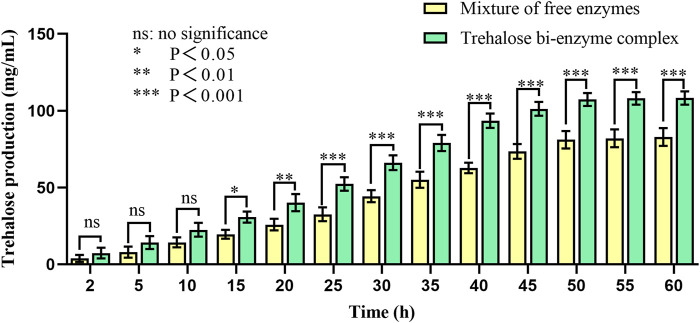
Kinetic comparison of trehalose production of the free enzyme mixture and double enzyme complex. Using maltodextrin with a concentration of 200 mg/mL as the substrate and the mixture of free enzymes as the control group, the mixture was transformed at 60°C for 60 h. The production of trehalose in the mixture of free enzymes and the bi-enzyme complex was monitored by HPLC during this period.

The trehalose bi-enzyme complex was successfully assembled under the aforementioned optimum conditions to detect the effect of trehalose production; taking the mixture of free enzyme MTSase and MTHase as the control group and paralleling three groups of experiments, the kinetic comparison diagram of trehalose production between free enzyme mixture and trehalose bi-enzyme complex within 60 h is drawn. As can be seen from Figure 7, the scaffold protein ScafCCR-mediated fusion enzymes Sase-*Cc*Doc and *Ct*Doc-Hase formed a bi-enzyme complex with a significantly better effect than the free enzyme mixture. In the beginning, the amount of trehalose produced in the bi-enzyme complex was higher than that in the free enzyme mixture; over time, the trehalose produced by the free enzyme mixture was 62.75 ± 3.4 mg/mL at 40 h, while the trehalose produced by the bi-enzyme complex was 93.57 ± 4.7 mg/mL, which reached 1.5 times of the free enzyme mixture and maintained an increasing trend, and the reaction was completed at 50 h. Therefore, the assembled trehalose bi-enzyme complex had the potential advantage of enhancing trehalose production.

The assembly effect was verified and characterized, the trehalose bi-enzyme complex with full use of the enzyme system was obtained, and the utilization and catalytic efficiency of the enzyme was improved. By comparing the ability of trehalose production by the trehalose bi-enzyme complex and free enzyme mixture, we found that with the passage of action time, the amount of trehalose produced by bi-enzyme complexes reached 1.5 times that of the free enzyme mixture at 40 h and maintained an increasing trend. Trehalose prepared by free enzymes has a poor cascade effect and long substrate action distance, resulting in low utilization and waste of double enzymes MTSase and MTHase. Therefore, the trehalose bi-enzyme complex formed by the assembly has the potential advantage of increasing trehalose production.

## 4 Discussion

Through the natural multi-enzyme configuration of cellulosomes, this paper aims to study the use of the artificial scaffold protein as a medium to complete the *in vitro* assembly of a bi-enzyme complex. Multiple enzymes can be co-immobilized by the scaffold proteins, significantly improving the catalytic efficiency of the reaction system (You et al., 2012; Ahmadi et al., 2021; Ivarsson et al., 2021). Therefore, the utilization of the scaffold protein as a means for multi-enzyme assembly emerges as a viable option. In this study, the artificial scaffold protein ScafCCR was employed alongside fusion enzymes Sase-*Cc*Doc and *Ct*Doc-Hase, resulting in successful construction and assembly through the specific interaction of cohesin–dockerin *in vitro*. In the beginning, we constructed two plasmids that directly fuse dockerin proteins at the C-terminal of the enzyme (the dockerin protein contains a natural linker), namely, MTSase-*Cc*Doc and MTHase-*Ct*Doc. We found that MTSase-*Cc*Doc was successfully expressed and enzyme activity was detected, but MTHase-CtDoc cannot detect enzyme activity. It is important to note that the enzyme activity of synthetic complexes is influenced by various factors, including inter-enzyme distance, enzyme orientation, and the overall architecture of the multi-enzyme system (Bayer et al., 2004; Lin et al., 2014; Smith et al., 2017). The unsuccessful construction of the fusion enzyme may be due to the linker (Wriggers et al., 2005; Kavoosi et al., 2007; Rozycki, 2016) or the spatial folding problem caused by the fusion of two protein domains (Arai, 2021). After we changed the rigidity, flexibility, and length of the linker between the enzyme MTHase and the dockerin protein *Ct*Doc, the constructed strain still did not detect any obvious enzyme activity. We then attempted to fuse the docking protein into the N-terminal of the enzyme and found that *Ct*Doc-MTHase was successfully expressed and had enzyme activity, which may be due to the disordered configuration of the fusion enzyme formed before the position adjustment or the formation of the configuration hindering the active site, resulting in no obvious enzyme activity (Cohen et al., 1969; Hong et al., 2018). The discovery of this construction problem also provides an opportunity for the application of cellulosomes in other enzyme fields in the future. The structure of the designed artificial scaffold protein was simulated using a Swiss model and processed using PyMOL software. From Supplementary Figure S1, it can be observed that the spatial structure of two cohesion proteins, *Ct*Coh and *Cc*Coh, is mainly shown as a β-folded double sheet form. The analysis of the amino acid composition of the two cohesins showed that hydrophobic amino acids were the main amino acids, which was also consistent with the previous reports (Bayer et al., 2008). The polar amino acids pointed out in the report are located on the outside, which plays a role in providing the corresponding recognition site of dockerin. Through the optimization of enzyme production conditions, the study determined the OD_600_ values of the fusion enzymes Sase-*Cc*Doc and *Ct*Doc-Hase to be 3.5 and 3.0, respectively. Additionally, the final concentration of IPTG was 0.1 mM, the induction temperature was 27°C, and the induction time was 16 h. The crude enzyme activities of the broken fermentation broth were approximately 12.9 U/mL and 15.8 U/mL, respectively. The growth of the recombinant strains affects enzyme production and activity after expression (Kim et al., 2021). When the strain is in the logarithmic growth stage, adding an inducer for fermentation can not only increase enzyme production but also improve enzyme activity (Hansen et al., 1998; Einsfeldt et al., 2011). On the premise of determining the optimal growth amount, adding IPTG can make it bind to the repressor protein, change its conformation, make it not interact with the target protein, and promote the high-efficiency expression of the target protein (Gomes et al., 2020). When the amount of the IPTG inducer added is higher than 0.4 mM, it will inhibit the growth of recombinant strains and reduce enzyme activity because IPTG itself is toxic. If the concentration exceeds the optimum, it will kill some bacteria, produce inclusion bodies, and reduce the content of soluble protein (Zheng et al., 2011; Kielkopf et al., 2021). Temperature not only affects the physical properties of the fermentation broth but also affects the properties of protein and the reaction rate of enzymes (Prentice et al., 2020). In addition, the enzyme activity of the fermentation broth gradually decreases, which may be since the temperature is too high, which leads to the easy aging of the strain and affects its growth and metabolism, thereby inhibiting its fermentation and enzyme production (Wang et al., 2016; Costa-Silva et al., 2019).

Under the optimum conditions, the target proteins Sase-*Cc*Doc, *Ct*Doc-Hase, and ScafCCR obtained by fermentation, expression, purification, desalination, and concentration were assembled *in vitro*. The experimental comparison between Native-PAGE and SDS-PAGE was used to verify the assembly effect of the trehalose bi-enzyme complex. Since the Native-PAGE electrophoresis process was affected by multiple factors such as protein morphology and charge, the electrophoresis bands of each protein are different from the SDS-PAGE electrophoresis bands separated only by molecular weight. The separation of Native-PAGE was not based on the labeled molecular weight of the protein but depended on the charge and natural structural state of different proteins (Nicke et al., 1999; Zheng et al., 2007). In addition to what was previously mentioned, Native PAGE is currently one of the key methodologies for studying non-denaturing proteomes because the mild nature of its electrophoresis process allows proteins to retain their full conformation and biological activity, which is advantageous for the advancement of subsequent experiments (Saravanan and Jocelyn, 2004; Alu’datt et al., 2019; Roehrkasse et al., 2021). Under non-denaturing conditions, protein migration is related to protein charge, protein shape, and protein molecular weight. Therefore, the molecular weight of the target protein cannot be accurately estimated. Although the map of Native-PAGE is ideal, more research is needed on whether protein complexes in protein extracts are denatured and whether interacting proteins still exist. The successful assembly of the trehalose bi-enzyme complex is shown in Figure 3A by the correspondence between a single band in Native-PAGE and three bands in SDS-PAGE in the same lane. In the research on the interaction between dockerin and cohesin, Ca^2+^ concentration is explored as an important factor. Temperature and pH can affect the activity of fusion enzymes, and Ca^2+^ can promote the folding of docking proteins to form stable tertiary structures (Rincon et al., 2003). The trehalose bi-enzyme complex is controlled in a neutral environment, Ca^2+^ concentration is 2–10 mM, and the effect of module assembly *in vitro* at 37 °C is the best, according to the study of module assembly under pH, temperature, and Ca^2+^ concentration. At a concentration of 15–25 mM Ca^2+^, it is evident from the degree of changes in protein bands that the docking effect between dockerins and cohesins gradually deteriorates, possibly due to the formation of Ca^2+^ precipitation in PBS buffer, resulting in incomplete self-assembly. The Ca^2+^ binding site is located in the docking protein domain, and Ca^2+^ can not only stabilize the structure of the docking protein but also stabilize the three-dimensional superstructure of the multi-enzyme complex (Hamberg et al., 2014). The report of Bule et al. (2018) is consistent with the research results of this paper that when Ca^2+^ concentration changes from 0.5 mM to 50 mM, the interaction of cohesion–dockerin is the most obvious. If the concentration of Ca^2+^ is too low or too high, it will severely limit the self-assembly of the cellulosome outside the cell, and intracellular self-assembly requires a lower concentration of Ca^2+^. Through the adsorption of the CBM module on ScafCCR and microcrystalline cellulose, the affinity pull-down experiment in Figure 3B showed that the scaffold protein carried the assembled fusion enzyme combined with microcrystalline cellulose and appeared in the precipitation part, which further verified the assembly effect of the trehalose bi-enzyme complex. Due to the residual unassembled fusion enzymes in the supernatant during the assembly process, the assembly efficiency needs to be further optimized (Gao et al., 2019; Yang et al., 2022). The gray value was analyzed by ImageJ software, and the standard curve was drawn after quantification. After calculation, the assembly and docking efficiency of ScafCCR and fusion enzymes Sase-*Cc*Doc and Hase-*Ct*Doc was approximately 70.16% and 65.94%, respectively. The structure, enzymatic properties, and ability to produce trehalose of the assembled trehalose bi-enzyme complex were analyzed. The docking fraction of *Cc*Doc and *Cc*Coh was 93.458 and that of *Ct*Doc and *Ct*Coh was 91.336 by combining molecular docking simulation and the CD spectrum. In general, *Cc*Doc*-Cc*Coh bonding generated three hydrogen bonds with an excellent binding effect, which was consistent with the conclusion that the effect of type I dockerin–cohesin derived from *C. cellulolyticum* was one of the strongest protein interactions in previous studies (Fierobe et al., 1999; Fierobe et al., 2001; Mechaly et al., 2001; Zverlov et al., 2008). The scaffolds of ScafCCR before and after assembly were found to have significant structural differences by performing circular dichroism. The main chain of ScafCCR before assembly was β-folded, while the trehalose bi-enzyme complex after assembly was partially α-helical. The α-helix secondary structure of dockerin could be observed from molecular docking simulation, and when dockerin–cohesin bonding formed strong hydrogen bonds, it will change the secondary structure of ScafCCR, which is the reason why there will be two configurations after assembly. For the assembled trehalose bi-enzyme complex, temperature and pH will not only affect its enzyme activity but also affect its efficiency. The optimum temperature is 70°C, and the optimum pH is 5.5. The half-life at the optimum temperature of 70°C is 28 h, and when the pH is maintained at 5.5–6.5, the relative enzyme activity of the trehalose bi-enzyme complex can be maintained at more than 90%. The assembly effect was verified and characterized, and the trehalose bi-enzyme complex with full use of the enzyme system was obtained, and the utilization and catalytic efficiency of the enzyme was improved. By comparing the ability of trehalose production by the trehalose bi-enzyme complex and free enzyme mixture, we found that over time, the amount of trehalose produced by the free enzyme mixture was 62.75 ± 3.4 mg/mL at 40 h, while the amount of trehalose produced by the bi-enzyme complex was 93.57 ± 4.7 mg/mL, which reached 1.5 times of the free enzyme mixture and maintained an increasing trend, and the reaction was completed at 50 h. Trehalose prepared by free enzymes has a poor cascade effect and long substrate action distance, resulting in low utilization and waste of free enzymes MTSase and MTHase. Inspired by the configuration of the cellulosome, the composition of multi-enzyme complexes can effectively solve this problem. The assembly of multi-enzyme complexes is to enhance the activity of cascade enzymes through substrate channels, and the substrate channel effect can greatly improve the catalytic effect of high-level enzymes and maintain the stability of enzymes (Zhang, 2011; You et al., 2012; Wheeldon et al., 2016; Tsitkov and Henry, 2019; Jiang et al., 2021). Therefore, the trehalose bi-enzyme complex formed by the assembly has the potential advantage of increasing trehalose production. The specific interaction between cohesin and dockerin is mainly used for cellulose degradation in existing technologies, with only a small amount applied to enzymes other than cellulase. The construction of an artificial scaffold protein and fusion enzymes as well as the assembly of a trehalose bi-enzyme complex using the specific interaction of cohesin–dockerin are all introduced in this article. This approach offers a novel perspective for enhancing enzyme production. To apply the scaffold protein-mediated bi-enzyme complex to trehalose production, more experiments are needed to prove the feasibility, including elucidating the deep mechanism of the multi-enzyme complex, searching for new cohesins and dockerins, the linker between fusion enzymes, the rational design of high-efficiency enzyme complexes, the role of cyclodextrin glycosyltransferse (CGTase), and whether the multi-enzyme complex can be recycled according to immobilization.

## Data Availability

The datasets presented in this study can be found in online repositories. The names of the repository/repositories and accession number(s) can be found in the article/Supplementary Material.
