# Supplementary material for: In vitro assembly of the trehalose bi-enzyme complex with artificial scaffold protein
Source: Front Bioeng Biotechnol. 2023 Aug 29;11:1251298. doi: 10.3389/fbioe.2023.1251298 (PMC10497880; doi:10.3389/fbioe.2023.1251298)
Supplement: Supplementary file 1 [file Table1.DOCX]

**Frontiers in Bioengineering and Biotechnology**

**Supplementary Material**

***In vitro* assembly of trehalose bi-enzyme complex with artificial scaffold protein**

Xiangyi Wang,^1,2^ Yi Jiang,^1,2^ Hongling Liu,^1,2^ Xinyi Zhang,^1,2^ Haibo Yuan,^1,2^ Di Huang,^1,2^ Tengfei Wang*^1,2^

^1^ State Key Laboratory of Biobased Material and Green Papermaking (LBMP), Qilu University of Technology (Shandong Academy of Sciences), Jinan 250353, Shandong, People’s Republic of China

^2^ Key Laboratory of Shandong Microbial Engineering, School of Bioengineering, Qilu University of Technology (Shandong Academy of Sciences), Jinan 250353, Shandong, People’s Republic of China

***Corresponding Author**

**Tengfei Wang -** School of Bioengineering, Qilu University of Technology, Jinan, CHINA

Emails: [wangtengfeisci@163.com](mailto:wangtengfeisci@163.com)

**Table S1 Primer sequence used in this study**

| Primers | Primer sequence (5’-3’) | Restriction site |
| --- | --- | --- |
| pET28a-F | GAAGGAGATATACCATGGGCAGCAG |  |
| pET28a-R | TTTAGAGGCCCCAAGGGGTTATGCT |  |
| ScafCCR-F | CAAATGGGTCGCGGATCCATGACAACAACAGGCGGC  CAG | *Bam*H Ⅰ |
| ScafCCR-R | TGGTGGTGGTGGTGGTGCTCGAGTTGTGTGCCCGGAT  CAATTG | *Xho* Ⅰ |
| Sase-*Cc*Doc-F | GGACAGCAAATGGGTCGCGGATCCATGGTGATATCAGCAACCTACAGATTA | *Bam*H Ⅰ |
| Sase-*Cc*Doc-R | TCTCAGTGGTGGTGGTGGTGGTGCTCGAGTTGAATGCTCAGCAGTGCTTTT | *Xho* Ⅰ |
| Hase-*Ct*Doc-F | GCAAATGGGTCGCGGATCCATGTTTTCGTTCGGTGGA  AAT | *Bam*H Ⅰ |
| Hase-*Ct*Doc-R | TGGTGGTGGTGGTGGTGCTCGAGATTTTTATACGGCAGTGTATCAATT | *Xho* Ⅰ |
| *Ct*Doc-F | CAAATGGGTCGCGGATCCATGTATGGCGATGTTAATGAT | *Bam*H Ⅰ |
| *Ct*Doc-R | TTTGTGCTCGGTGTGCCCGGAACTTTATACGTATTTTTATACGGCAGTGT |  |
| Hase-F | TTCCGGGCACACCGAGCACAAAACTGTTTTCGTTCGGTGGAAATATTG |  |
| Hase-R | TGGTGGTGGTGGTGGTGCTCGAGTTCTAATTGATATACCCCAACACC | *Xho* Ⅰ |

* The previously constructed recombinant strain *E. coli*/ pET28a Hase-*Ct*Doc, no obvious enzyme activity was determined. So, using the synthesized pUC57-Hase-*Ct*Doc plasmid as the template, the gene fragments of Hase and *Ct*Doc were amplified by primers Hase-F/Hase-R and *Ct*Doc-F/*Ct*Doc-R.

**Table S2 Plasmids used in this study.**

Malto-oligosyltrehalose trehalohydrolase(MTSase). NCBI Reference Sequence: WP_011278268.1

Malto-oligosyltrehalose synthase(MTHase). NCBI Reference Sequence: WP_088899914.1

Due to codon optimization, the synthetic sequences differ from the natural sequences.

synthetic sequences:ScafCCR(GenBank: OQ630934, OQ630935), MTSase-*Cc*Doc(GenBank: OQ630936, OQ630937), *Ct*Doc-MTHase(GenBank: OQ630938, OQ630939)

natural sequences: ScafCCR gene (GenBank: CAC34384.3, MH049738.1), MTSase gene (GenBank: D64128.1), *Cc*Doc gene (GenBank: MH049736.1), MTHase gene (GenBank: CP020363.1), CtDoc gene (GenBank: L06942)

| Plasmids | Characteristics | Source | | |
| --- | --- | --- | --- | --- |
| pET28a (+) | *E. coli* expression vector, KanaR,  *T7/lac* promoter, f1origin | | Lab stock | |
| pUC57-ScafCCR | To obtain artificial scaffolding genes | | | Sangon Biotech  (Shanghai, China) |
| pUC57-Sase-Ccdoc | Used to obtain the target gene of treY/*Cc*Doc | | | Sangon Biotech (Shanghai, China) |
| pUC57-Hase-Ctdoc | Used to obtain the target gene of treZ/*Ct*Doc | | | Sangon Biotech  (Shanghai, China) |
| pET28a-ScafCCR | Used for expression of artificial scaffolding genes ScafCCR, KanaR, *T7/lac* promoter,  f1 origin | | | This work |
| pET28a-Sase-Ccdoc | Used for expression of fusion protein Sase-*Cc*Doc, KanaR, *T7/lac* promoter, f1 origin | | | This work |
| pET28a-Ctdoc-Hase | Used for expression of fusion protein *Ct*Doc-Hase, KanaR, *T7/lac* promoter, f1 origin | | | This work |

**Table S3 Enzymatic activity of fusion enzymes**

| Fusion enzyme | Specific activity(U/mg) | Activity (U/mL) |
| --- | --- | --- |
| Sase-*Cc*Doc | 178.64 | 12.9 |
| *Ct*Doc-Hase | 265.27 | 15.8 |

**Table S4** **Data analysis of the assembled fusion enzymes in Image J software**

| Enzyme | Area | Mean gray | OD | Total OD |
| --- | --- | --- | --- | --- |
| Sase-*Cc*Doc  (Lane 7 in Fig. 4B) | 18062 | 233.991 | 1.084134799 | 19581.64275 |
| *Ct*Doc-Hase  (Lane 7 in Fig. 4B) | 19986 | 226.988 | 0.959196063 | 19170.49251 |

**Fig. S1**

**
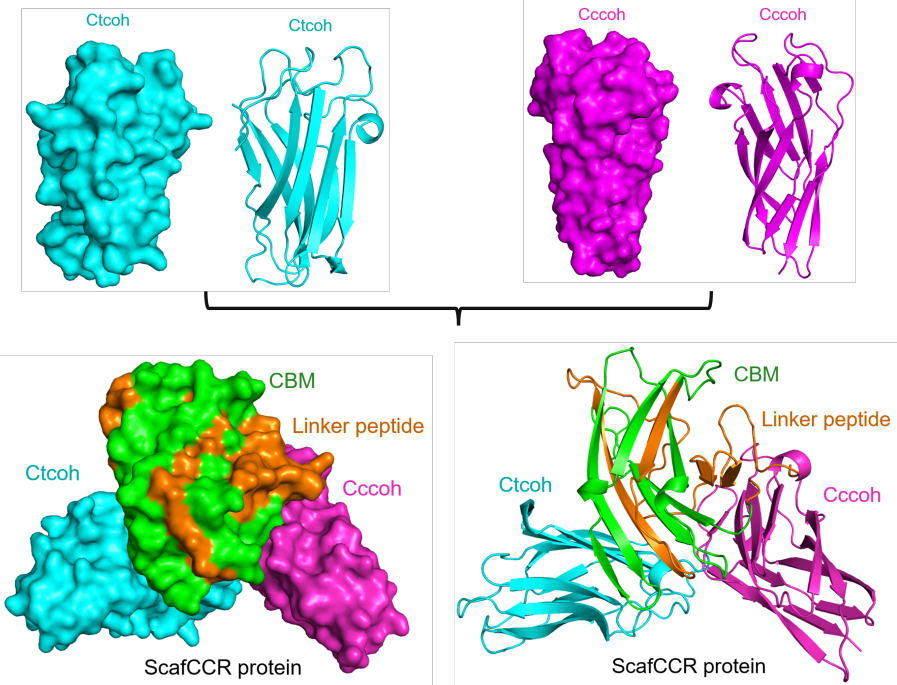
**

**
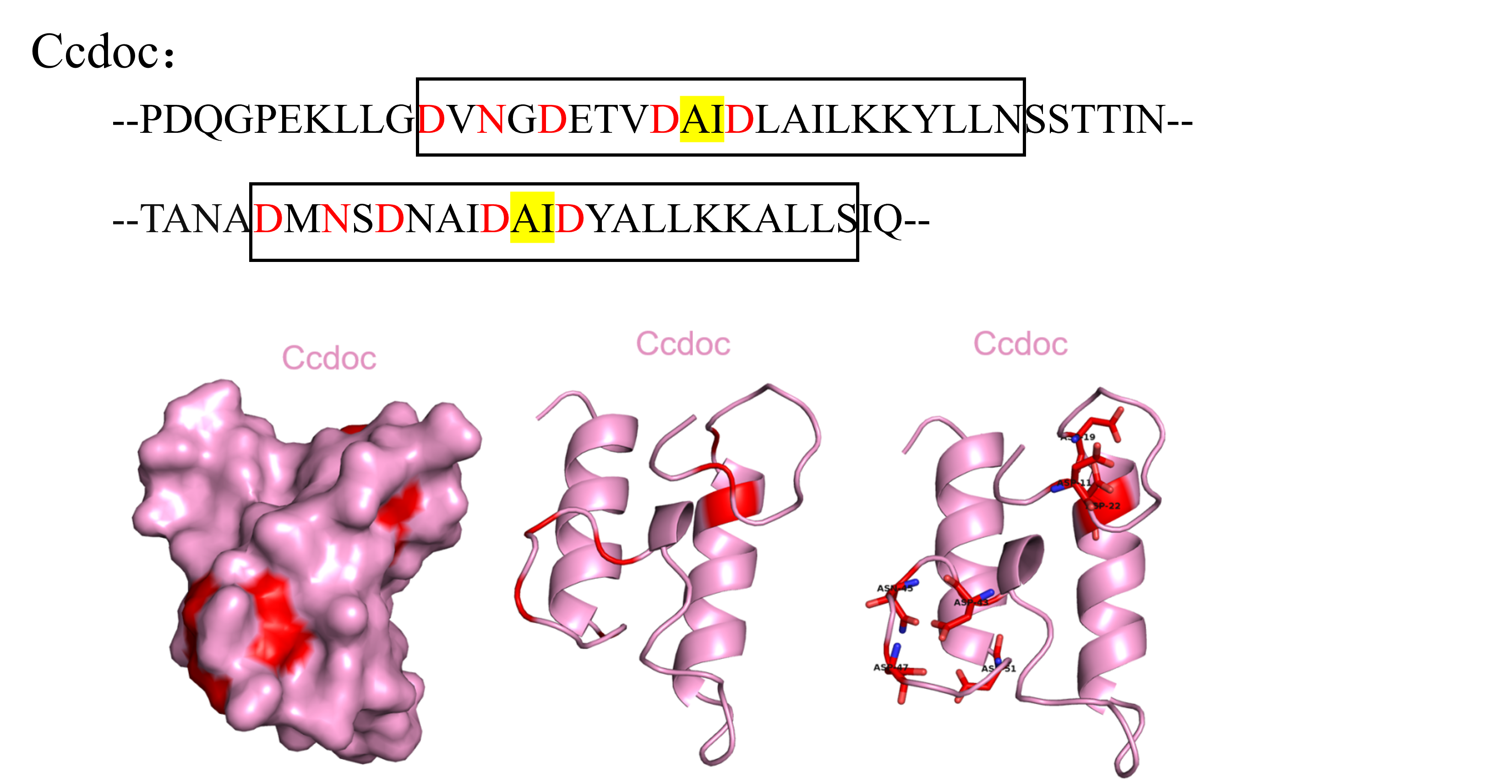

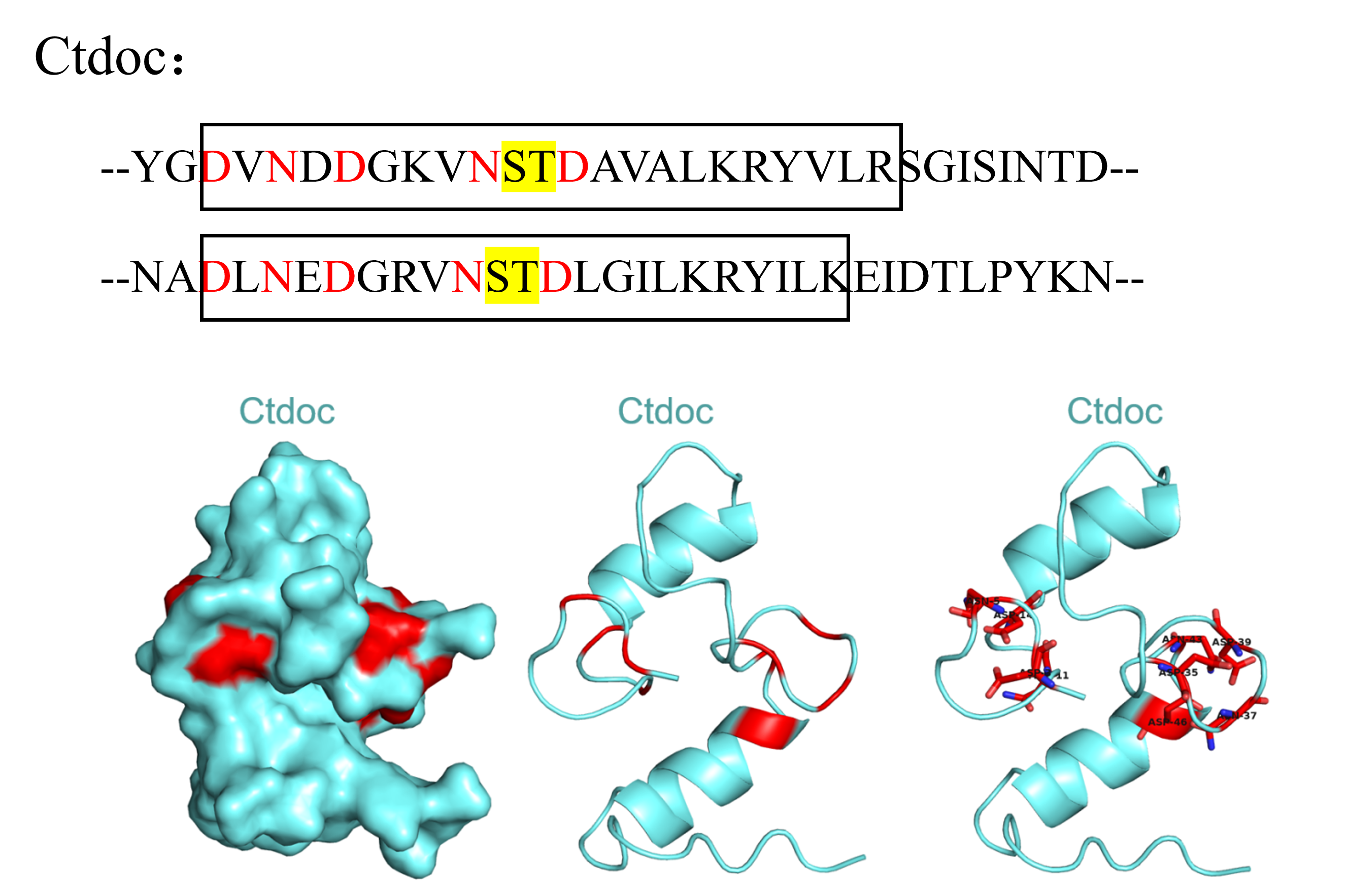
**

**Figure S1** Structural simulation diagram of the scaffold protein of ScafCCR and the dockerin protein of *Cc*Coh and *Ct*Coh. **A:** The scaffold protein containing *Ct*Coh, linker, CBM, linker and *Cc*Coh from N-terminal to C-terminal. which was obtained from Swiss-model and PyMOL software. **B and C:** The mimetic structure of dockerin *Cc*Doc and dockerin *Ct*Doc. And the red part is the binding of Ca^2+^.

**Fig. S2A**


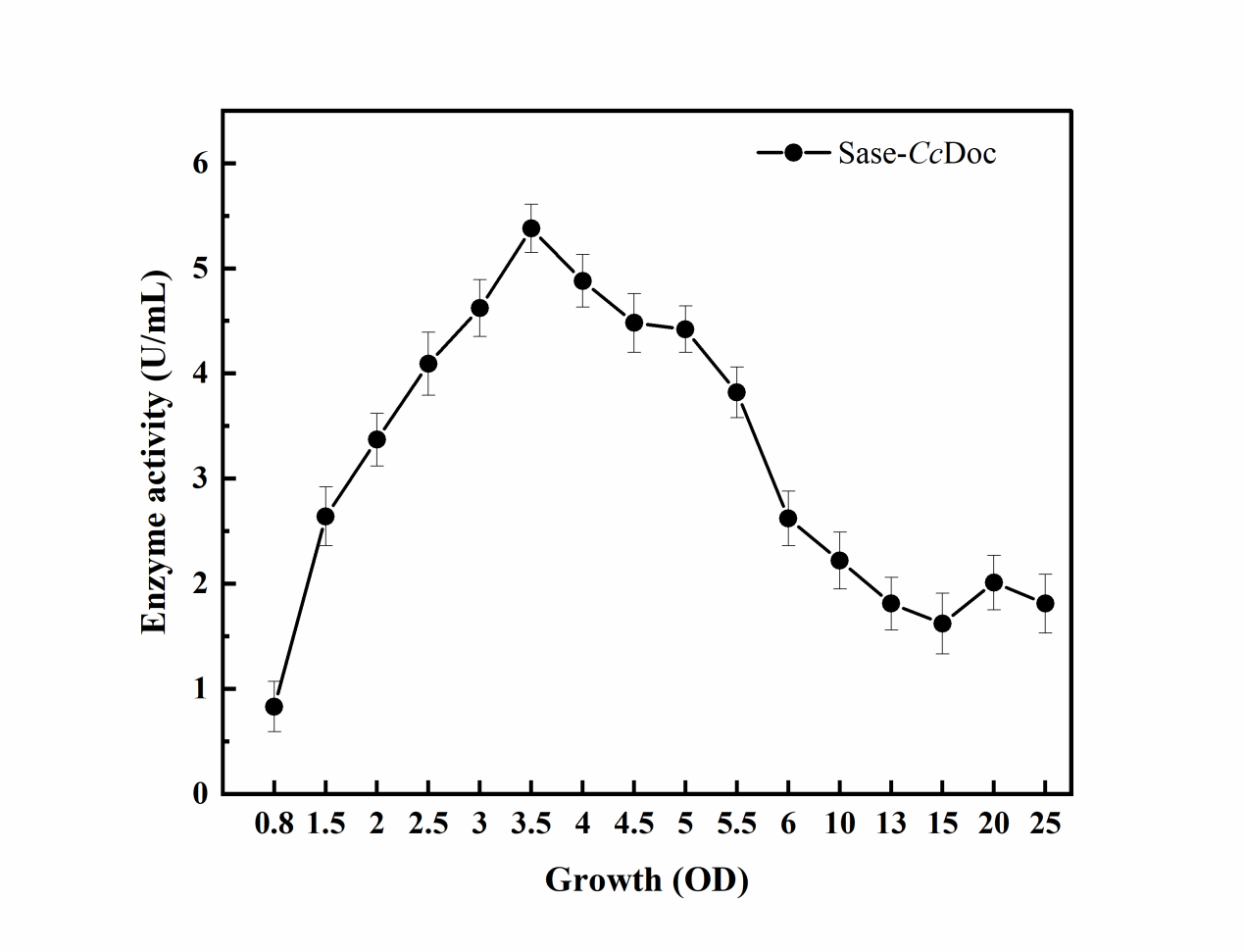


**Fig. S2B**


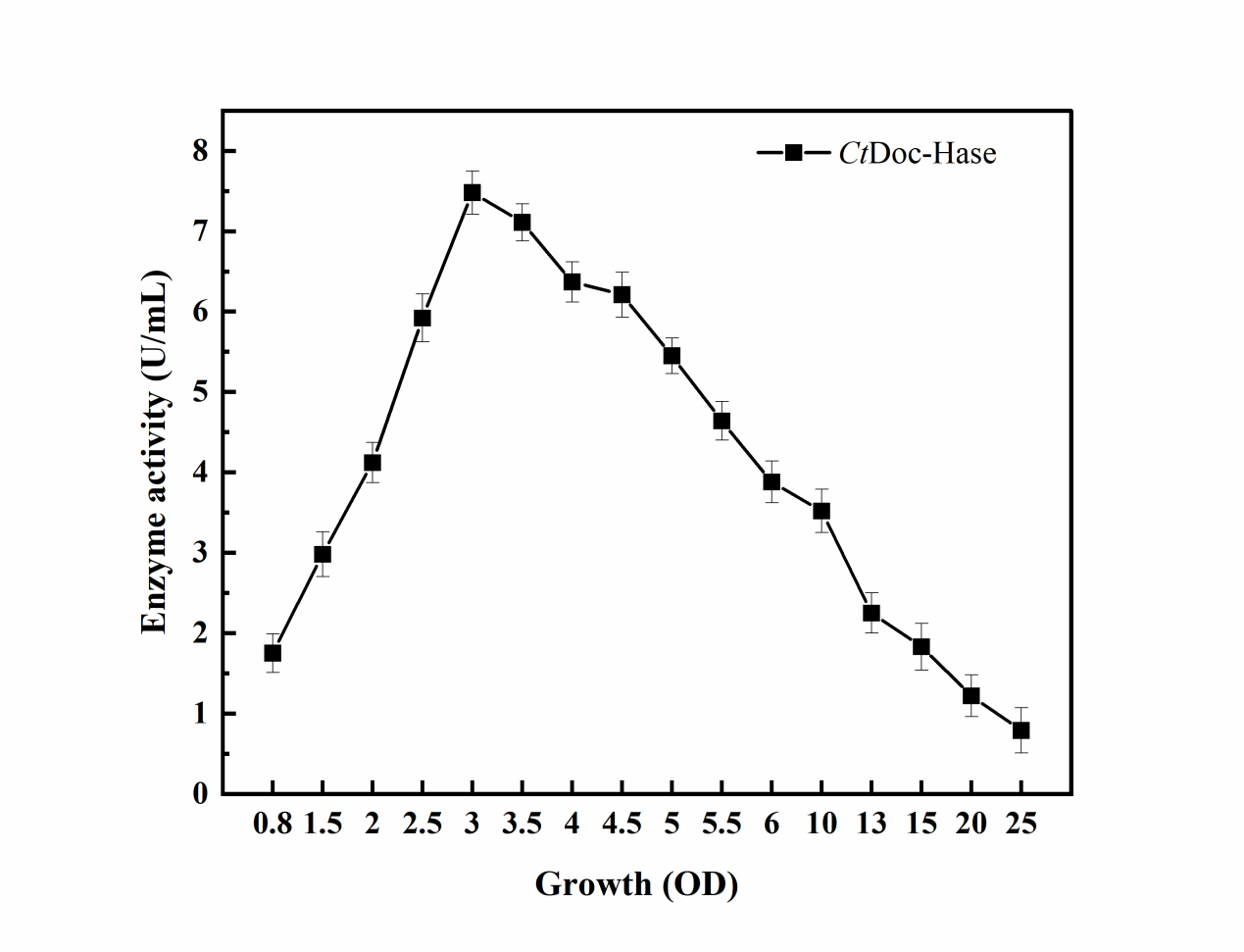


**Fig. S2C**


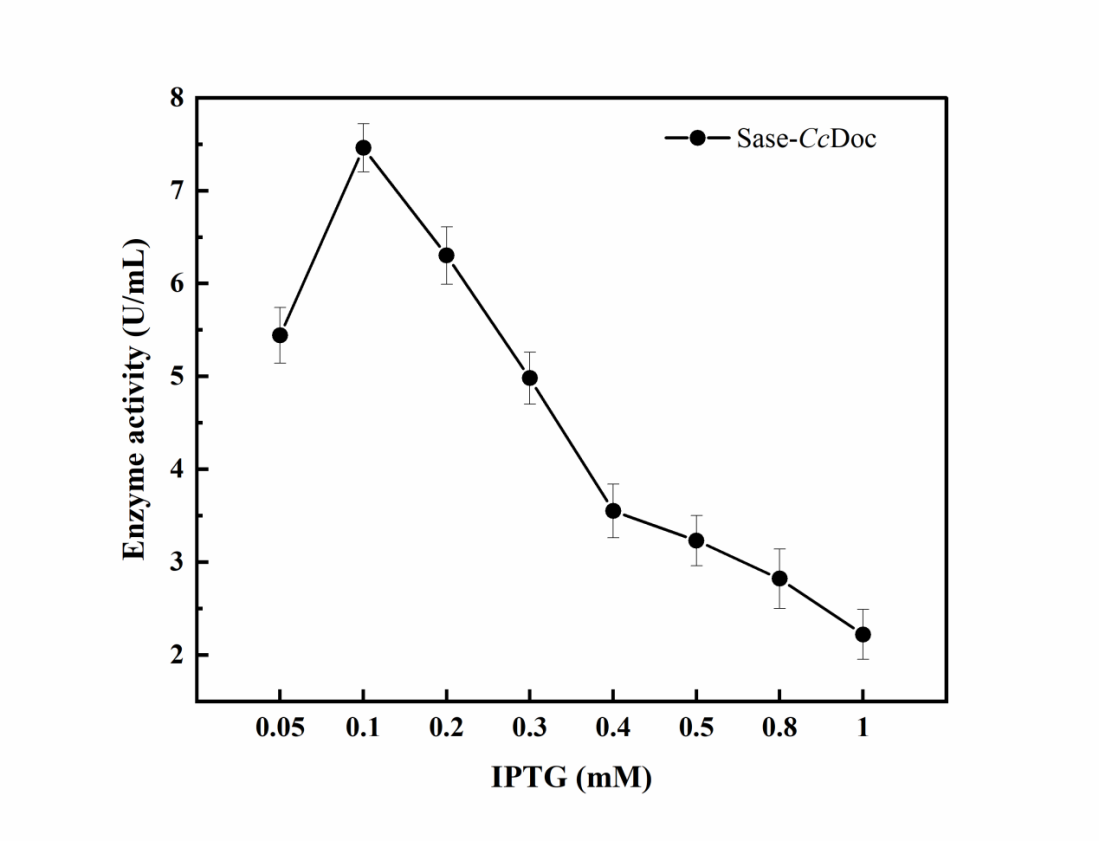


**Fig. S2D**


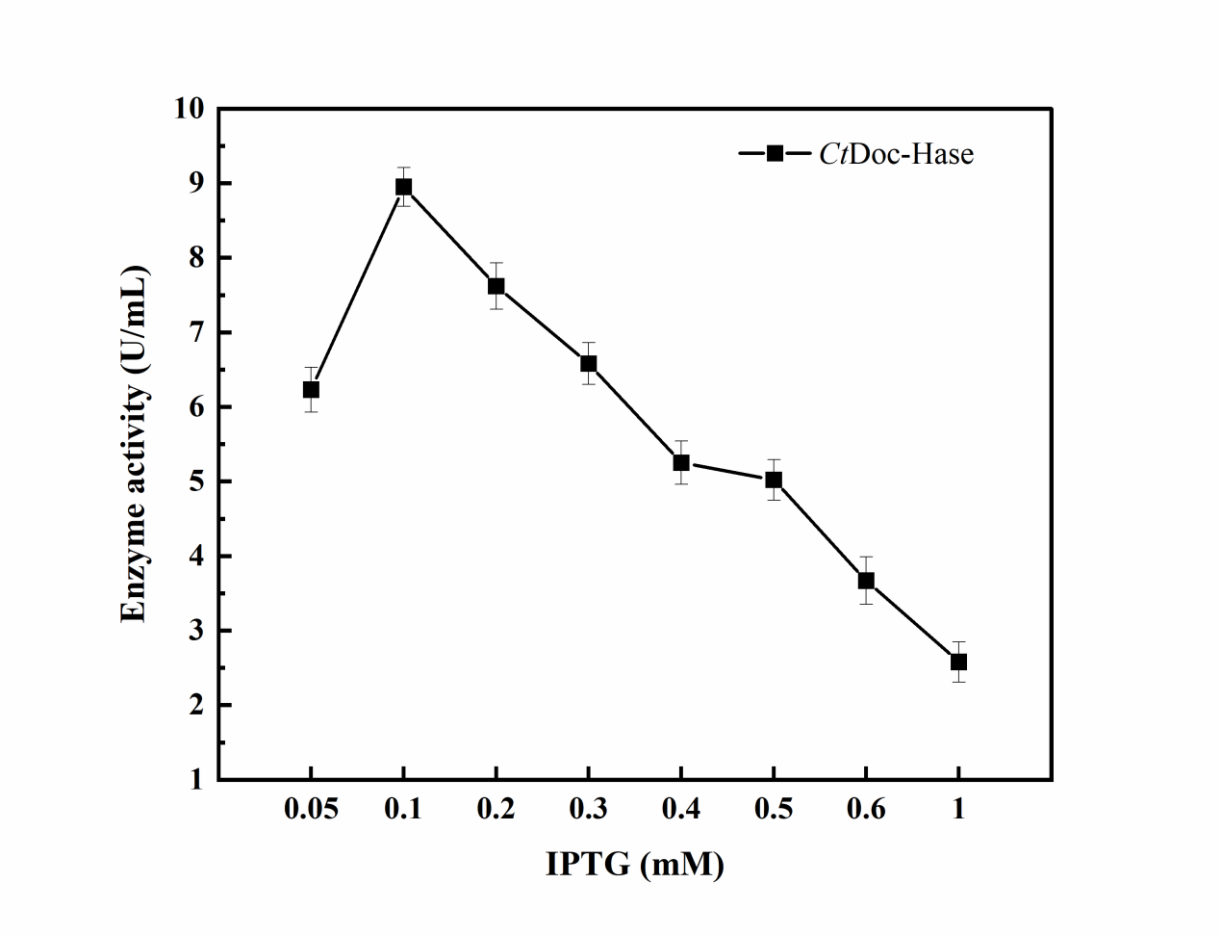


**Fig. S2E**


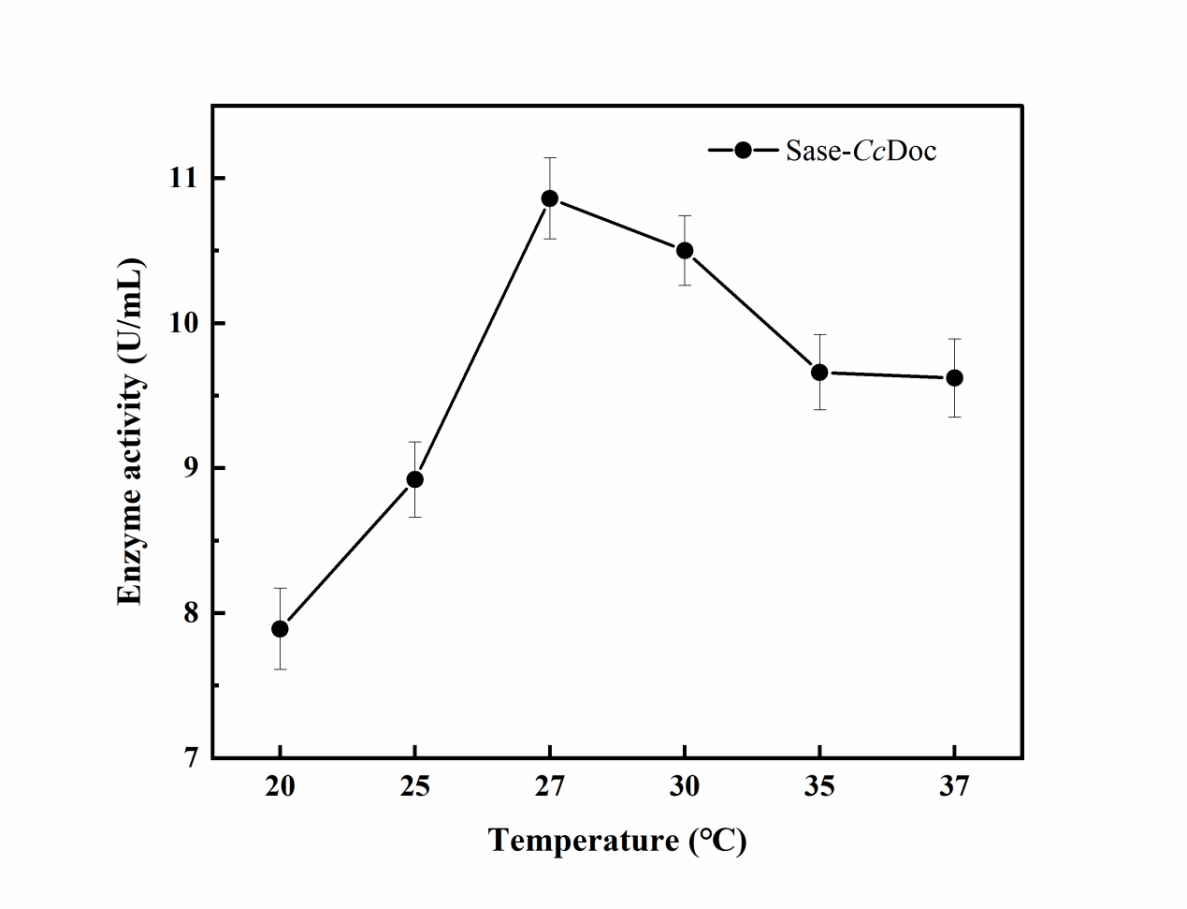


**Fig. S2F**


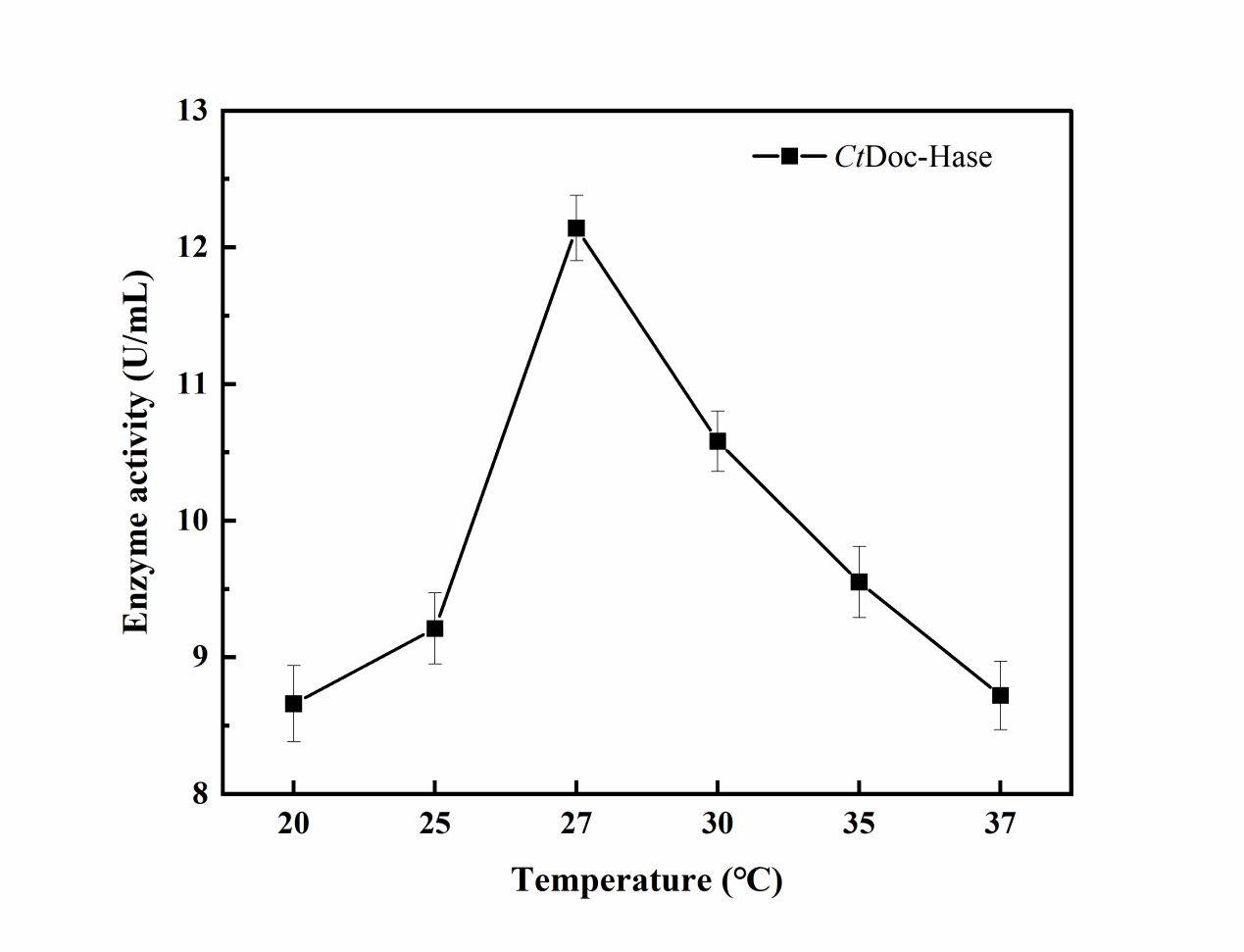


**Fig. S2G**


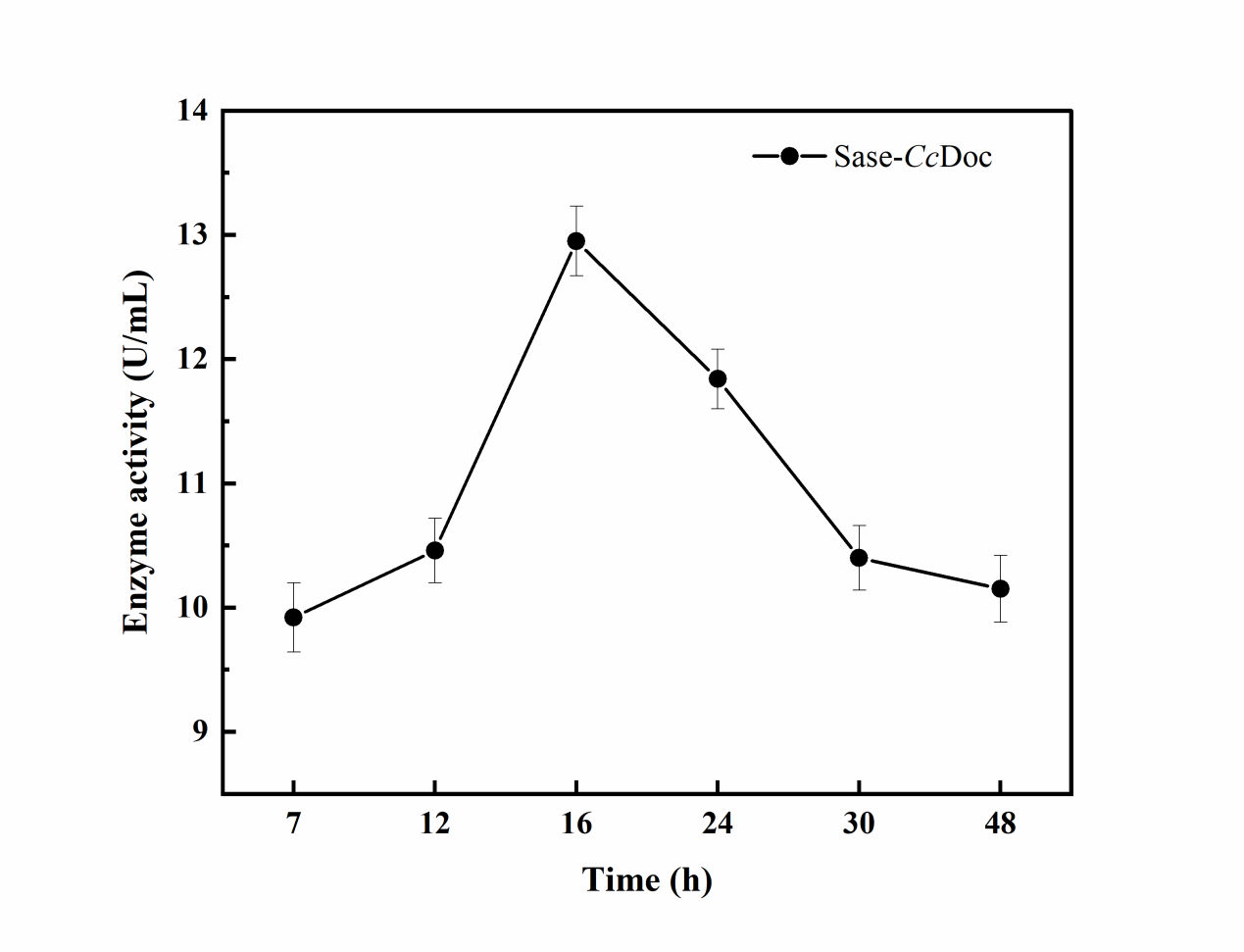
**Fig. S2H**


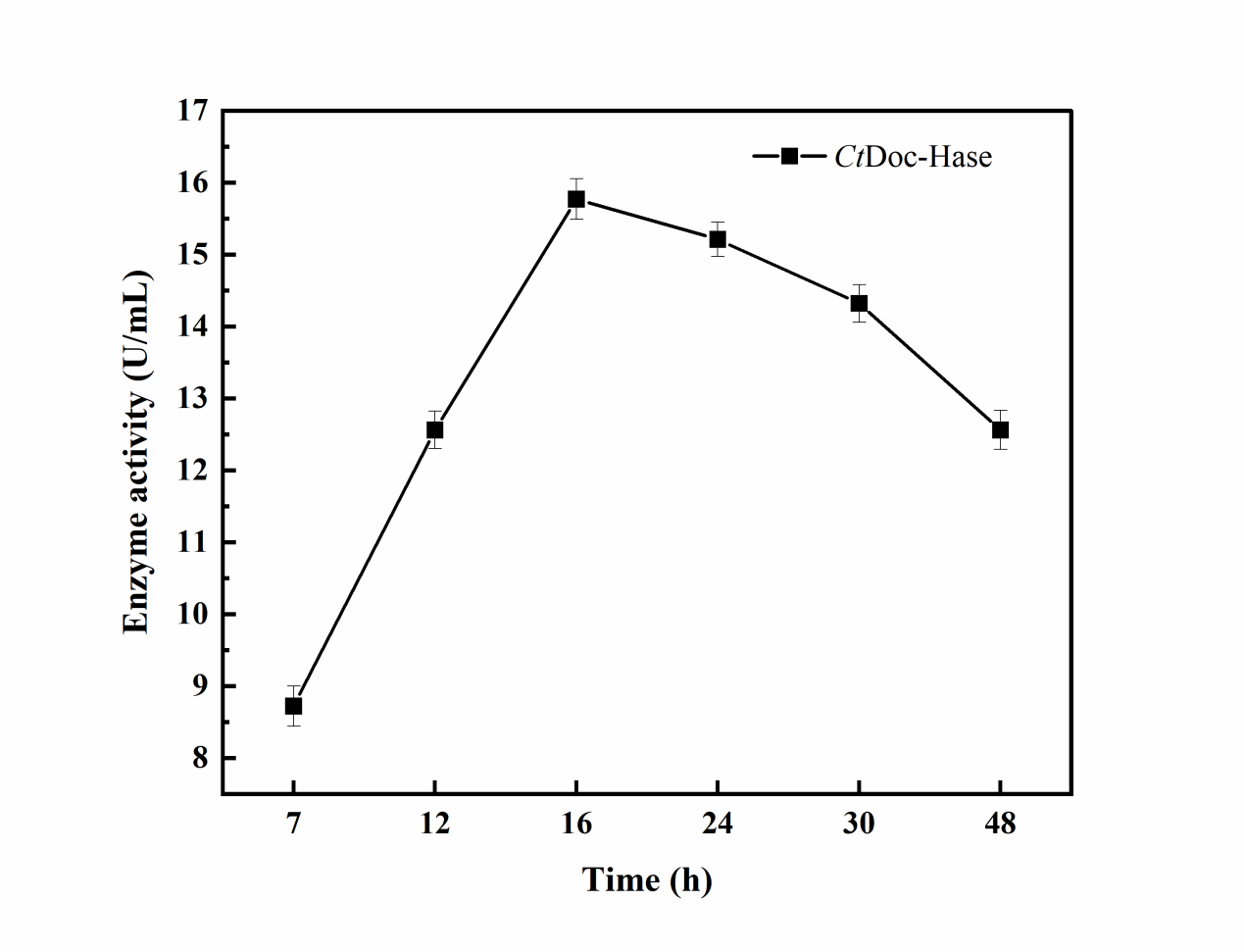


**Figure S2** Effects of growth amount, IPTG addition, induction temperature and induction time on enzyme production. **A:** Effect of growth amount of recombinant strain *E. coli*/pET28a Sase-*Cc*Doc on enzyme production. **B:** Effect of growth amount of recombinant strain *E. coli*/pET28a *Ct*Doc-Hase on enzyme production. **C:** Effect of IPTG addition of recombinant strain *E. coli*/pET28a Sase-*Cc*Doc on enzyme production. **D:** Effect of IPTG addition of recombinant strain *E. coli*/pET28a *Ct*Doc-Hase on enzyme production. **E:** Effect of induction temperature of recombinant strain *E. coli*/pET28a Sase-*Cc*Doc on enzyme production. **F:** Effect of induction temperature of recombinant strain *E. coli*/pET28a *Ct*Doc-Hase on enzyme production. **G:** Effect of induction time of recombinant strain *E. coli*/pET28a Sase-*Cc*Doc on enzyme production. **H:** Effect of induction time of recombinant strain *E. coli*/pET28a *Ct*Doc-Hase on enzyme production
